# The role of autophagy in the treatment of type II diabetes and its complications: a review

**DOI:** 10.3389/fendo.2023.1228045

**Published:** 2023-09-21

**Authors:** Xuan Zhao, Lu-Yao Bie, Dao-Ran Pang, Xiao Li, Long-Fei Yang, Dan-Dan Chen, Yue-Rui Wang, Yan Gao

**Affiliations:** ^1^ Institute of Pharmaceutical Research, Shandong University of Traditional Chinese Medicine, Jinan, China; ^2^ Tsinghua University-Peking University Joint Center for Life Sciences, School of Life Sciences, Tsinghua University, Beijing, China; ^3^ Innovative Institute of Chinese Medicine and Pharmacy, Shandong University of Traditional Chinese Medicine, Jinan, China; ^4^ College of Traditional Chinese Medicine, Shandong University of Traditional Chinese Medicine, Jinan, China

**Keywords:** Type II diabetes mellitus (T2DM), autophagy, complications, hypoglycemic agents, traditional Chinese medicine

## Abstract

Type II diabetes mellitus (T2DM) is a chronic metabolic disease characterized by prolonged hyperglycemia and insulin resistance (IR). Its incidence is increasing annually, posing a significant threat to human life and health. Consequently, there is an urgent requirement to discover effective drugs and investigate the pathogenesis of T2DM. Autophagy plays a crucial role in maintaining normal islet structure. However, in a state of high glucose, autophagy is inhibited, resulting in impaired islet function, insulin resistance, and complications. Studies have shown that modulating autophagy through activation or inhibition can have a positive impact on the treatment of T2DM and its complications. However, it is important to note that the specific regulatory mechanisms vary depending on the target organ. This review explores the role of autophagy in the pathogenesis of T2DM, taking into account both genetic and external factors. It also provides a summary of reported chemical drugs and traditional Chinese medicine that target the autophagic pathway for the treatment of T2DM and its complications.

## 1 Introduction

**Figure 1:**
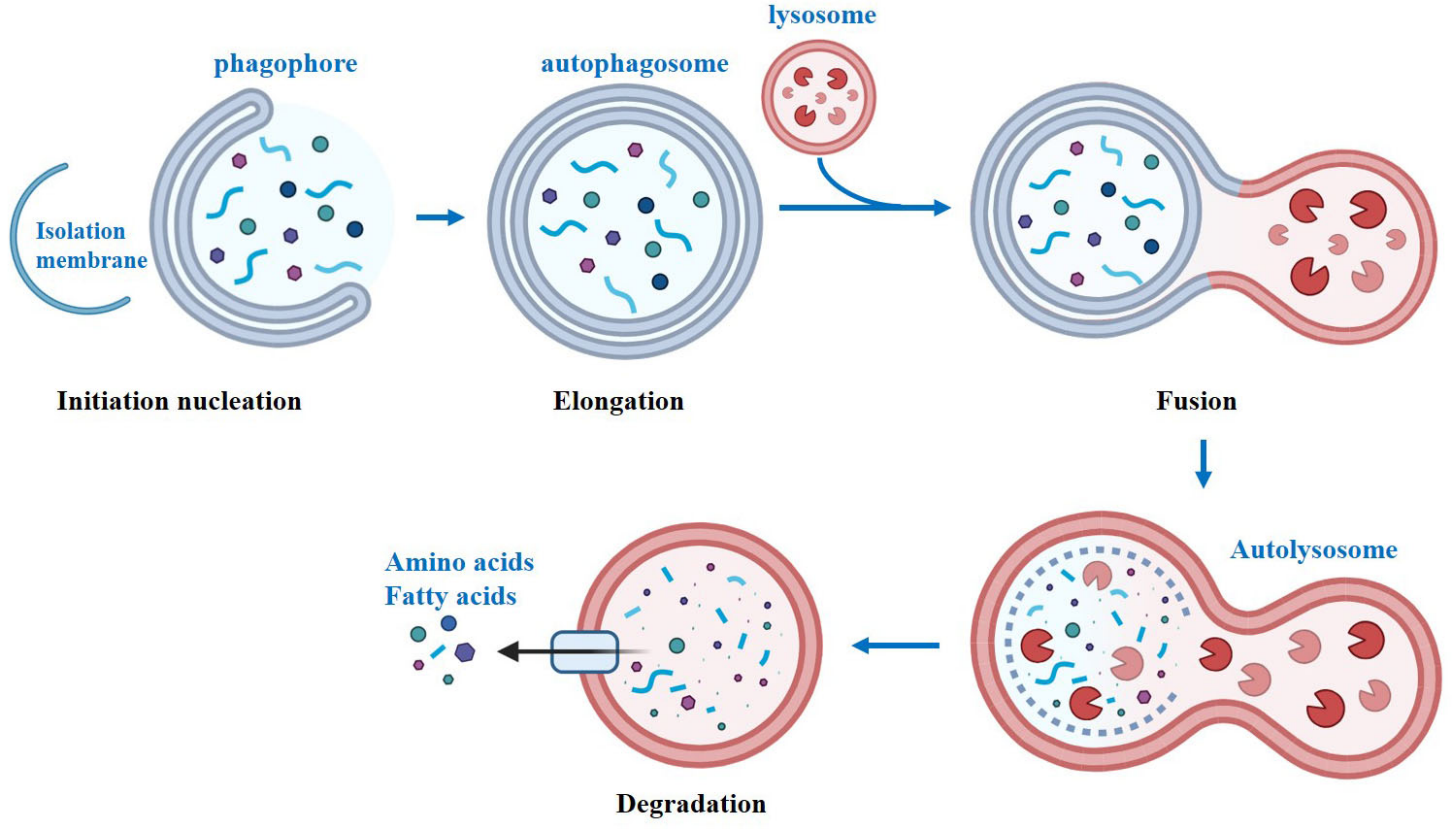
The classical process of autophagy.

Autophagy, also known as self-cleaning and self-eating, is a conserved biological process in eukaryotes (1). Autophagy can provide nutrients to maintain cellular function by breaking down macromolecules, organelles, proteins, and end products, under starvation conditions. Moreover, it also helps in maintaining cellular homeostasis by eliminating damaged organelles, misfolded proteins, and lipid droplets (2). There are two types of autophagy: selective and non-selective, which are triggered by different signals. While selective and non-selective autophagy respond to different signals, both pathways converge to initiate autophagy (3). In mammals, autophagy initiation is linked to the omegasome, a functional domain of the endoplasmic reticulum (ER) that is rich in the lipid phosphatidylinositol 3-phosphate (PI3P). Starting from the omegasome, the isolation membrane elongates into a cup-shaped structure and initiates the process of phagocytosing intracellular material (4). Overall, autophagy is a process that can be divided into four steps (**Figure 1**). The first step is initiation, where the ULK1-dominant protein complex triggers the formation of another important protein complex called PI3KC3-C1. These two complexes are then recruited to the phagophore assembly site, where they assist in the formation of autophagosomes. The second step is extension. ATG12 is activated by ATG7, leading to the formation of an ATG12-ATG5 complex. This complex then interacts with either ATG16 or ATG16L1 to form the ATG12-ATG5-ATG16L complex. Simultaneously, ATG7 facilitates the binding of phosphatidylethanolamine (PE) to LC3-I, resulting in the formation of LC3-II. The sequential assembly of these protein-protein and protein-lipid complexes enables the extension of the autophagosome bilayer. The third step involves the maturation of autophagosomes, where closed autophagosomes are formed. In the fourth step, autophagosomes fuse sequentially with lysosomes to form autophagic lysosomes. Subsequently, the contents are digested by lysosomal hydrolases, and the resulting metabolites become available for recycling. The identification of crucial genes for autophagy in yeast has revolutionized our comprehension of mammalian physiology and human pathophysiology. To date, over 35 autophagy-related genes have been identified in yeast (5). Among these genes, the 15 core genes necessary for starvation-induced autophagy and selective autophagy are highly conserved in mammals (6). Autophagy-associated gene mutations have been linked to numerous human diseases (7). During the progression of chronic diseases, the accumulation of damaged organelles, protein aggregates, lipid droplets, and aging cells has been observed, which is speculated to be related to the disruption of autophagy (8, 9).

**Figure 2:**
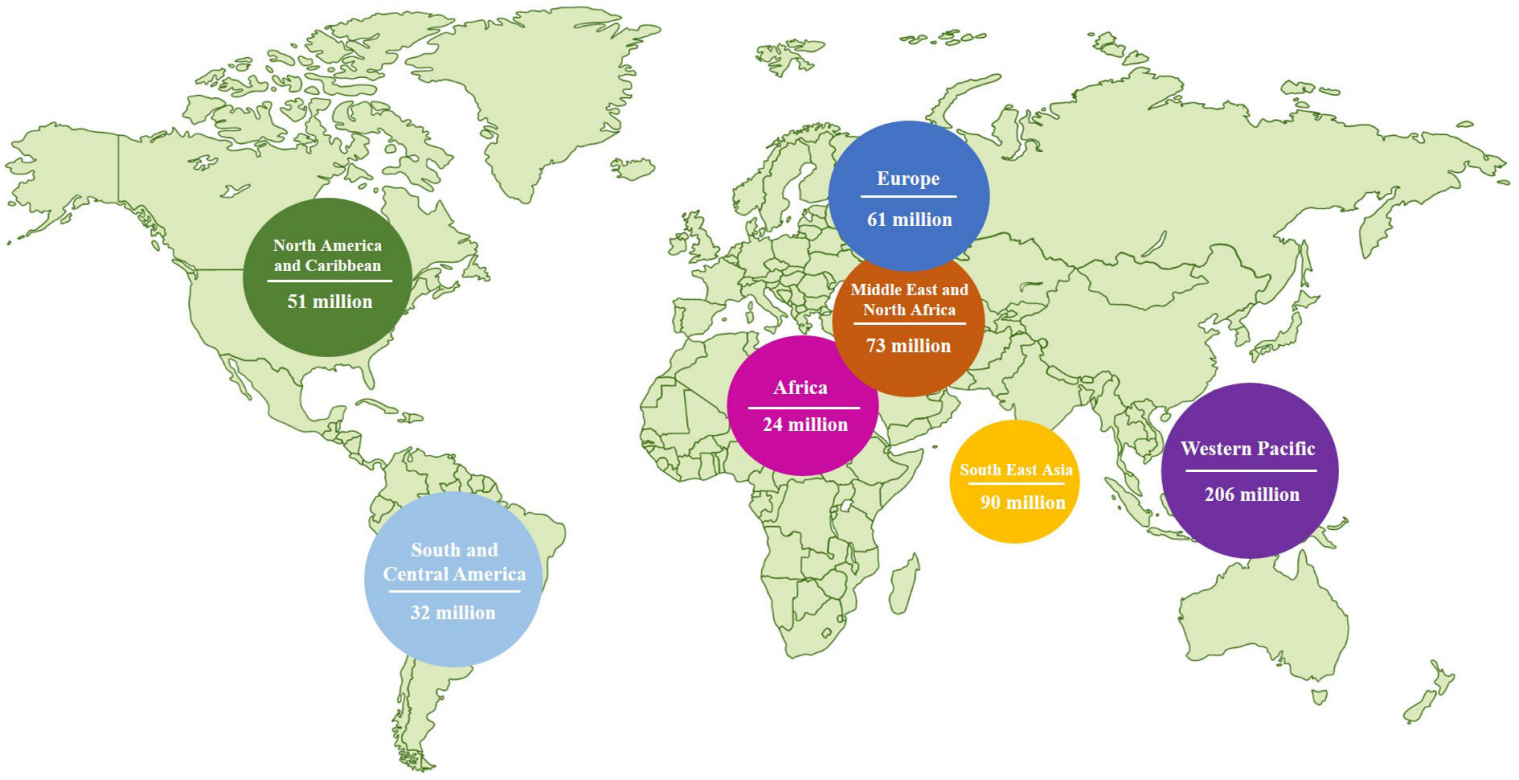
Diabetes around the world in 2021 (data was from IDF).

Diabetes mellitus (DM) is a metabolic disease characterized by chronic hyperglycemia. Type II diabetes mellitus (T2DM) is the most common type of DM, accounting for over 90% of diabetic patients (10). Typically, the elderly population is more susceptible to T2DM. However, due to improved living standards and changing lifestyles, the incidence of T2DM is increasing among children, teenagers, and young adults. According to data from the International Diabetes Federation (IDF), there are currently 537 million T2DM patients worldwide as of November 2021 (11) (**Figure 2**). The IDF predicts that this number will rise to 643 million by 2030 and 784 million by 2045 (11). The prevalence of diabetes in China is approximately 10%, with a total of 114 million diabetic patients, representing one-third of the world’s diabetic population (12). Insulin resistance (IR) is a characteristic of Type 2 Diabetes Mellitus (T2DM) where the efficiency of insulin to absorb and utilize glucose is reduced. This results in the compensatory secretion of more insulin, leading to hyperinsulinemia (13). The overuse of islet cells eventually exhausts the pancreas, reducing its ability to produce insulin and ultimately leading to diabetes mellitus (13). According to the United Kingdom Prospective Diabetes Study (UKPDS), around 50% of individuals with T2DM have impaired islet cells, with more than 90% of these individuals also experiencing Insulin Resistance (IR) (14). In addition to high blood sugar levels, diabetic patients are at an increased risk of developing various health complications. Prolonged high blood sugar can result in a decline in organ function and severe complications affecting the heart, blood, eyes, kidneys, nerves, and teeth. It can even cause serious cardiovascular disease, blindness, renal failure, and amputation (15). Diabetes mellitus has become a silent threat in modern life, significantly impacting people’s daily lives and reducing their quality of life. Diabetes mellitus imposes a significant psychological burden on patients and a substantial economic burden on both society and families (16).

**Figure 3:**
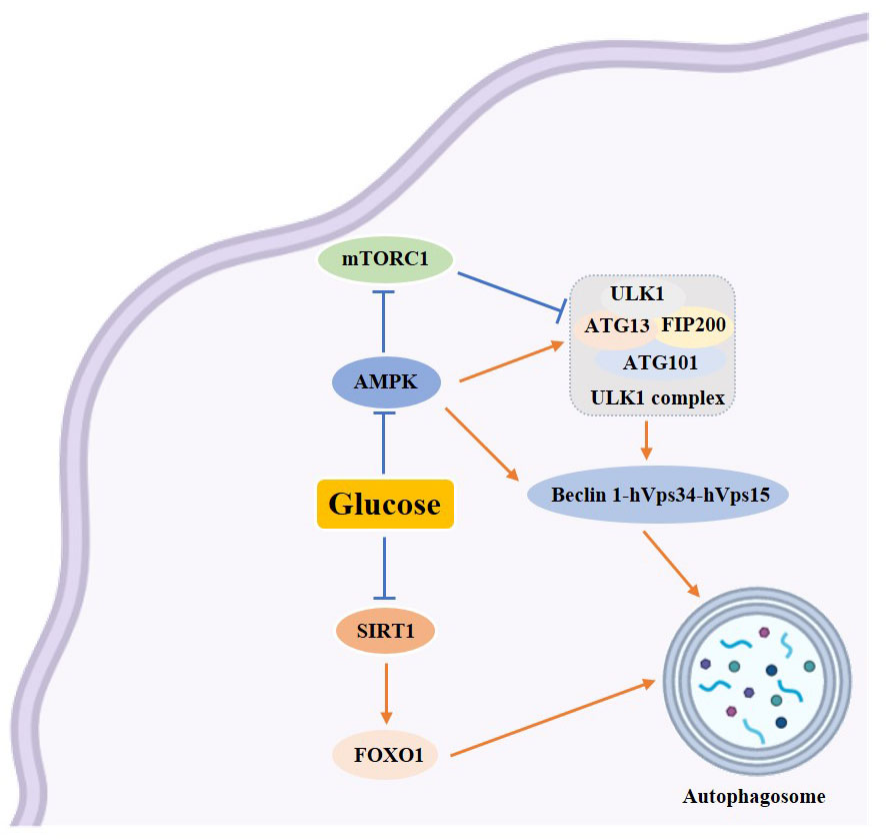
Schematic diagram of the relationship between autophagy and glucose metabolism. →: active; ⟞ :inhibit.

Some studies indicate that autophagy plays a significant role in the study of human diseases (2), particularly in glucose metabolism (17, 18) (**Figure 3**). Understanding the mechanism of autophagy is crucial in researching diabetes mellitus and its complications. This review examines the regulation of autophagy in diabetes and its complications, specifically focusing on the changes in autophagy caused by high-glucose and high-fat conditions. It also explores the effects of nutrient deficiencies and excesses on autophagy. Additionally, the review investigates how autophagy regulates endoplasmic reticulum stress and oxidative stress in diabetic states. Furthermore, it discusses the role of autophagy in the regulation of diabetic complications and explores how both chemical and traditional Chinese medicines target autophagy in the treatment of diabetes and its complications. This review’s aim is to provide some inspiration for the treatment of T2DM.

## 2 Overview of autophagy in diabetic states

**Figure 4:**
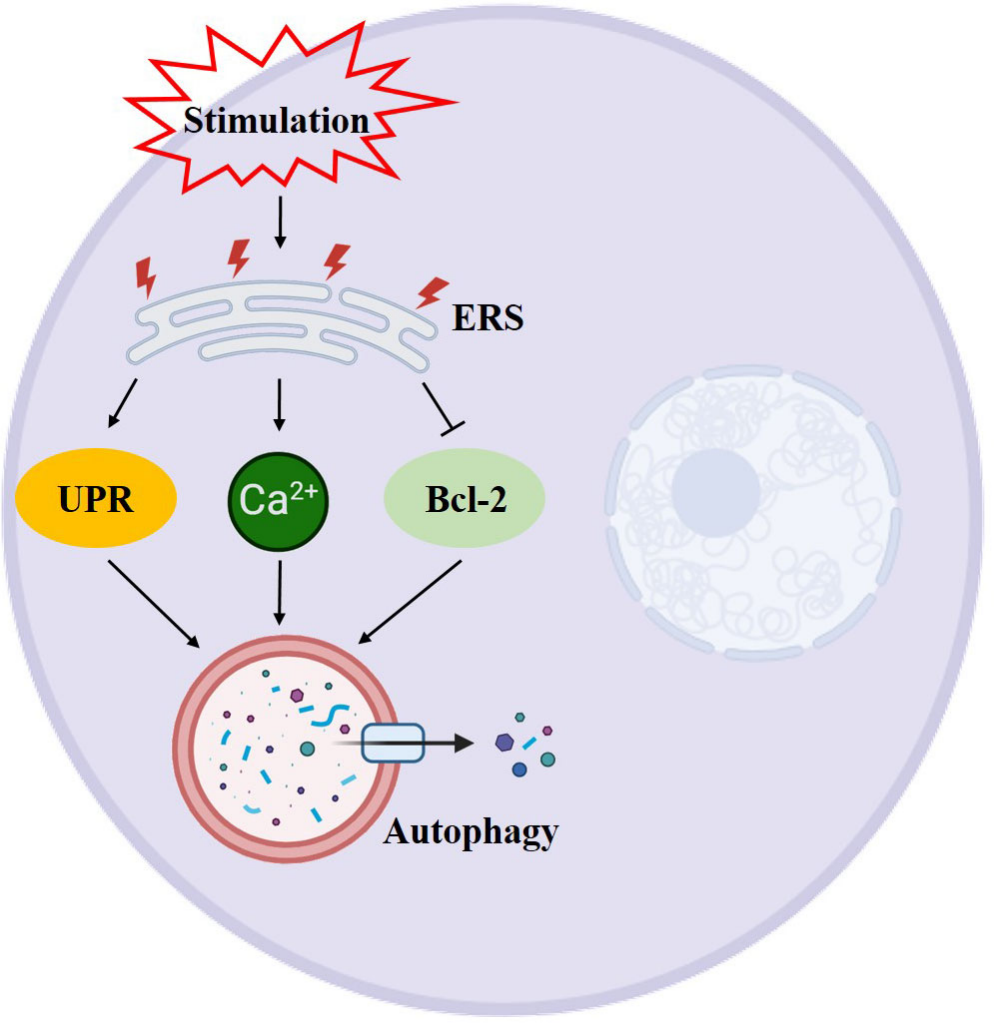
Schematic diagram of the relationship between ERS and autophagy. →: active; ⟞: inhibit.

Autophagy is intricately linked to glycolipid levels within the body, and it exerts a protective effect on pancreatic β-cells (19). Studies have shown that a prolonged high-glucose-high-fat diet can hinder autophagy while fasting and restricting calorie intake can activate it (20). Sustained hyperglycemia and excess free fatty acids can lead to an increase in mitochondrial oxygen consumption, resulting in an elevation of reactive oxygen species (ROS) and subsequent abnormal oxidative stress (21). Additionally, ROS can induce autophagy through various mechanisms, including the modulation of autophagy via the mTOR and MAPK signaling pathways, the regulation of autophagy by ROS through the PI3K/Akt, AMPK, and ERK, JNK signaling pathways, and the oxidation of ATG4 by ROS to regulate autophagy (22–24). To summarize, oxidative stress activates autophagy, which helps eliminate cellular damage caused by oxidative stress and maintain cellular homeostasis. Excessive levels of ROS can lead to endoplasmic reticulum stress, endoplasmic reticulum stress can also be induced by insulin resistance. Evidence suggests that insulin inhibits autophagy, while glucagon activates it (25). Insulin, the sole hormone responsible for lowering glucose levels in the body, is synthesized within the endoplasmic reticulum. The endoplasmic reticulum holds significant importance within the cell as it is involved in various processes such as protein synthesis, transport, folding, and degradation. Maintaining homeostasis within the endoplasmic reticulum is crucial for the proper functioning of β-cells. When pancreatic β-cells are stimulated by prolonged high glucose levels, it disrupts homeostasis and leads to an accumulation of unfolded and misfolded proteins in the endoplasmic reticulum of the cells. In order to ensure cell survival, these misfolded proteins need to be eliminated, triggering endoplasmic reticulum stress (26). When endoplasmic reticulum stress is induced by strong intracellular and extracellular stimuli, the endoplasmic reticulum has two mechanisms to deal with the accumulation of misfolded proteins. One is the unfolded protein response (UPS), and the other is endoplasmic reticulum associated degradation (ERAD). However, in cases where both UPS and ERAD, activated by endoplasmic reticulum stress, are insufficient to restore the endoplasmic reticulum to its normal state, autophagy becomes the last resort to restore endoplasmic reticulum homeostasis. Autophagic vesicles engulf the damaged endoplasmic reticulum, which is then transported to lysosomes for degradation. Studies have shown that ERS and dysregulated autophagy are interconnected features of diabetes in human pancreatic islet β-cells (26). Endoplasmic reticulum stress can restore the normal function of the endoplasmic reticulum through cellular autophagy. This process involves inducing cellular autophagy through unfolded proteins response (UPR) (27), promoting Ca^2+^ influx into the cytoplasm to induce cellular autophagy (28), and inhibiting Bcl-2 to induce endoplasmic reticulum autophagy (29) (**Figure 4**). The occurrence of autophagy has both positive and negative effects on the endoplasmic reticulum. Moderate autophagy can reduce endoplasmic reticulum swelling and alleviate the pressure caused by the accumulation of faulty proteins. However, excessive autophagy can result in cell death (30).

## 3 Correlation between autophagy and T2DM as well as its complications

**Figure 5:**
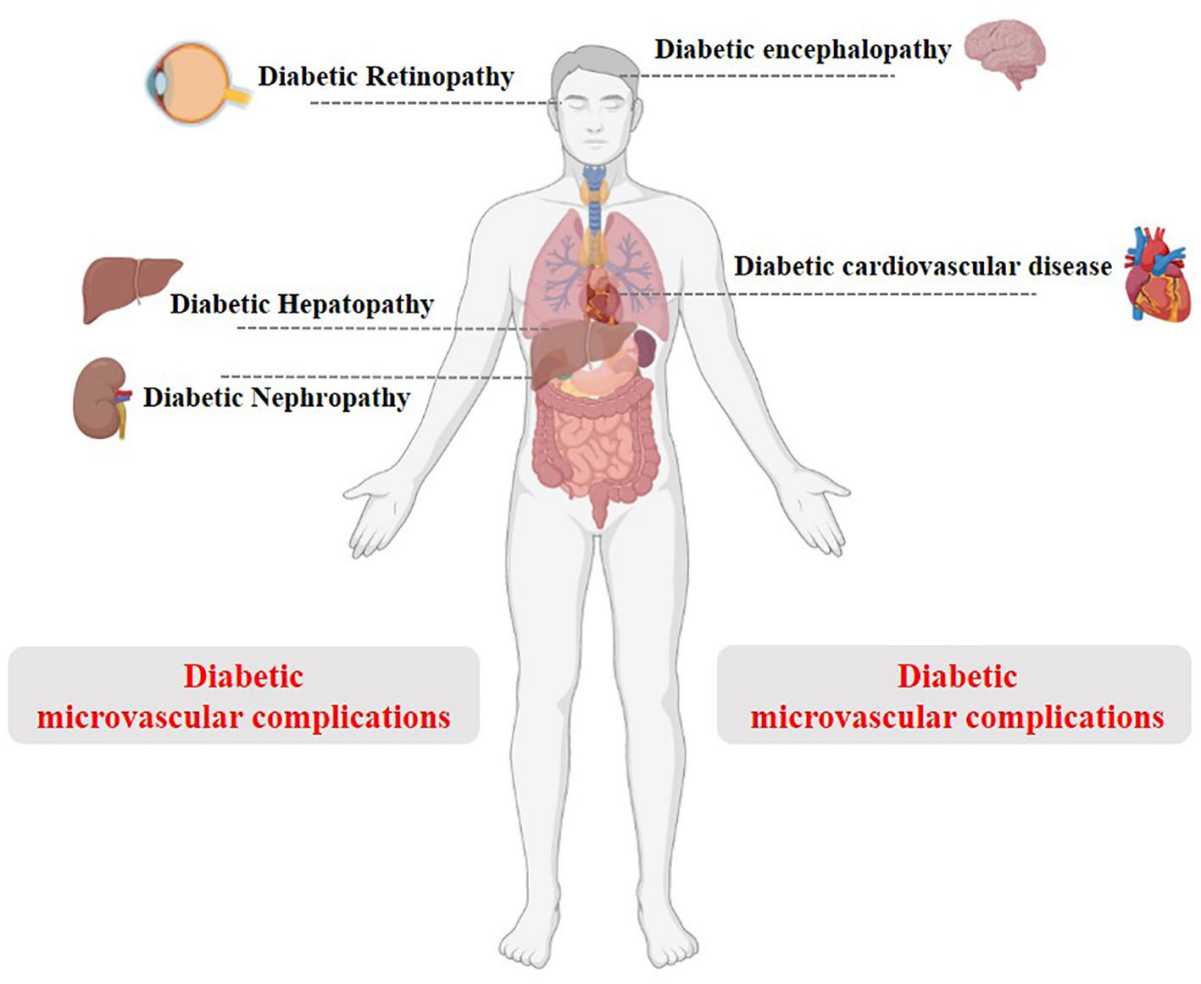
Schematic diagram of T2DM and its complications.

In recent years, there has been a growing body of research highlighting the significance of autophagy in the development of diabetes and its associated complications (**Figure 5**). This section aims to shed light on the role of autophagy in the progression of diabetes and its complications and treatment.

### 3.1 Autophagy and β islet cells

T2DM is a progressive disease that initially presents as insulin resistance. In order to achieve the desired blood glucose level, increased insulin secretion by pancreatic β-cells is required to compensate (13). However, over time, the pancreatic β-cells become overwhelmed by the increased workload and begin to fail, resulting in reduced insulin secretion to compensate for insulin resistance (13, 31). The mechanisms that regulate pancreatic β-cell fate in T2DM are not yet fully understood. However, studies have shown that autophagy plays a crucial role in maintaining normal islet architecture (32). Human pancreatic cells with impaired autophagy regulation have been observed to have an increased association with β-cell dysfunction and failure (33–37). On the other hand, increased autophagy has been found to improve insulin resistance (38–41), and it may serve as a survival mechanism for β-cells (42, 43).

This section delves into the significance of autophagy in pancreatic β-cells, particularly in the context of T2DM. The mTORC1 is a natural inhibitor of autophagy and is crucial for promoting the growth and survival of islet β-cells during the early stages of insulin resistance (44). However, excessive mTORC1 activity due to a high-calorie diet can disrupt autophagy (45). Conversely, inhibiting mTORC1 can enhance autophagy and aid in the removal of misfolded proteins and damaged organelles (46). Bartolomé A et al. (47) demonstrated that chronic overactivation of mTORC1 in a mouse model (β- tsc2 -/-) led to increased pancreatic β-cell death and impaired autophagy. In a separate study, Matthew R. Brown et al. (48) found that negative regulation of REV-ERBα may improve β-cell function under glucotoxicity by enhancing autophagy. This was achieved by investigating the link between the core circadian clock nuclear receptor REV-ERBα, autophagy, and β-cell failure. Rui Liang et al.’s research (49) discovered that in both healthy and diabetic pancreases, the autophagy regulators LC3 and p62/SQSTM1 were found to be expressed more significantly in β cells than in non-β endocrine cells. Additionally, the expression of LC3 and p62/SQSTM1 was significantly reduced in T2DM patients and was inversely correlated with HbA1c levels, suggesting that the autophagic ability of β-cells is impaired as the disease progresses. Tanima Chatterjee et al.’s research (50) discovered that inhibiting autophagy regulation in pancreatic β-cells resulted in apoptosis, ultimately leading to cell death. In a separate study, pancreatic β-cell lines and human islets were exposed to high levels of glucose, leading to an accumulation of autophagosomes, impaired mitochondria, and increased mTOR expression (51). These findings suggest that high glucose levels block autophagic flux and lead to cell death. However, when treated with rapamycin, the changes in autophagic flux as well as glucose-induced cell death were reversed.

Several chemical drugs have also demonstrated good regulation of autophagy to treat diabetes. *Liraglutide*, a GLP-1 analog (52), has demonstrated effective regulation of autophagy for the treatment of diabetes. Liraglutide has been found to upregulate autophagy mediated by FoxO1, which helps to improve pancreatic β-cell injury (53). Additionally, Liraglutide has been reported to protect INS-1 cells from apoptosis induced by high glucose levels and significantly increase cellular autophagy (54). These findings suggest the potential of Liraglutide in targeting autophagy to prevent β-cell apoptosis. Another drug, *Exendin-4*, a GLP-1 receptor agonist, has also been found to prevent Tac-induced islet injury by activating autophagosome clearance to reduce autophagosome burden (55). *Metformin*, on the other hand, inhibits MIN6 β cell proliferation and promotes apoptosis through AMPK-dependent and autophagy-mediated mechanisms (56). Additionally, studies have shown that *vitamin B6* can protect RIN-m5f cells from apoptosis caused by high glucose levels through the mTOR pathway-mediated autophagy (57).

In addition to chemical drugs, traditional Chinese compounds and extracts have been found to improve the course of T2DM by regulating autophagy. *Yunpi Heluo decoction (YPHLD)* has been reported to regulate the SIRT1-FoxO1 signaling pathway in skeletal muscle, improve lipid metabolism, increase autophagy levels, and attenuate insulin resistance, potentially making it an effective treatment for diabetes (58). *Xiaokeping (XKP)* has also shown potential in protecting islet β-cells from high glucose toxicity by inducing mTOR-mediated autophagy and reducing pancreatic β-cell apoptosis (59). Additionally, *Morus alba leaves ethanol extract* has been found to protect islet cells from dysfunction and death by inducing AMPK/mTOR-mediated autophagy (60). Finally, *Dioscin* has been shown to significantly attenuate insulin resistance in adipose tissue through the IRS-1/PI3K/Akt pathway (61). *Kaempferol* has been shown to activate autophagy through the AMPK/mTOR pathway, resulting in pancreatic β-cell protection in the treatment of T2DM (62). Similarly, *Silymarin*, a flavonoid found in the fruit of Silybum marianum, has been found to increase autophagy and protect INS-1 cells from TNFα or IL-1β-induced death. This is achieved through the activation of autophagy-dependent ERs, which help maintain cellular energy homeostasis (63).

**Figure 6:**
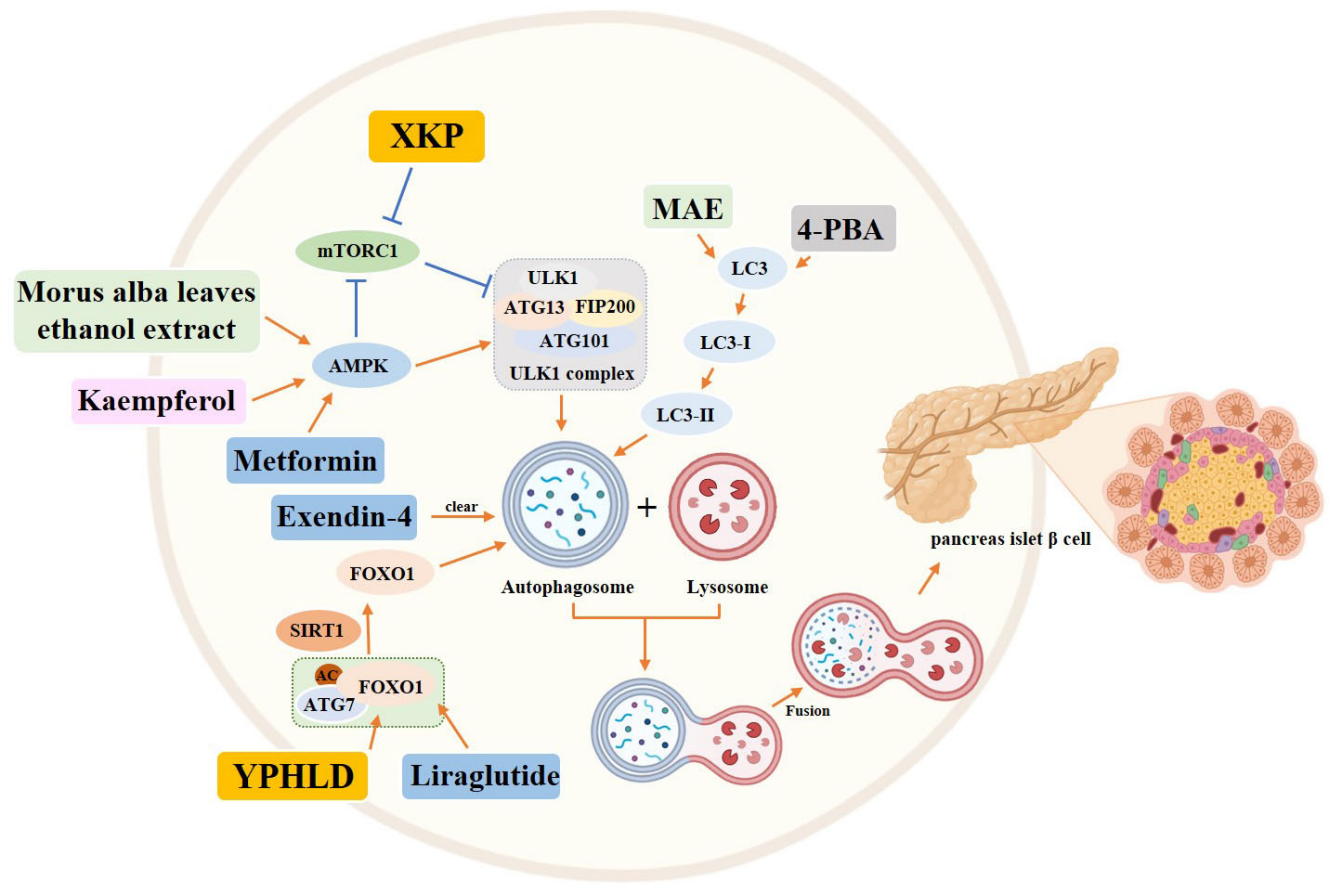
Role of autophagy in pancreatic islet β cells in the diabetic state. Yellow rectangle: Traditional Chinese compounds; Blue rectangle: Chemical drugs; Pink rectangle: Monomers from Chinese Herbal; Gray rectangle: Experimental Chemicals. →: activate: ⟞: inhibit.

In addition to drug therapy, certain experimental chemicals have shown promise in modulating autophagy to alleviate T2DM. Specifically, *4-Phenylbutyric acid* and *rapamycin* have been found to induce autophagy and increase the autophagic flux, ultimately preventing β-cell apoptosis (64) (**Figure 6**).

*Exercise* has been found to promote autophagy by activating the AMPK/PGC1α pathway, which can help reduce insulin resistance in mice (65).

This discovery suggests that increasing autophagy could potentially delay disease progression and maintain β-cell function in T2DM. Therefore, inducing autophagy in islet cells through exogenous drugs or other techniques could be a promising approach for preventing or treating T2DM.

### 3.2 Autophagy and diabetic nephropathy

The kidney is highly susceptible to diabetic microvascular injury due to prolonged high blood glucose levels (66–68). This can lead to cellular glucotoxicity and increased oxidative stress, resulting in irreversible kidney damage and end-stage renal disease, known as diabetic nephropathy (DN) (69). Diabetic nephropathy is a frequent complication of diabetes, affecting around 50% of diabetic patients and being the primary cause of death in those with type I and type II diabetes (66, 70–72).

Research indicates that traditional therapies are ineffective in slowing down the advancement of diabetic nephropathy (73–75). Additionally, there is mounting evidence that autophagy is a crucial factor in maintaining kidney health, preventing disease, and slowing the aging process. Autophagy has been shown to contribute to the development of DN (68, 76–79), and targeting it may have a positive impact on kidney function (80). This section will delve into the role of autophagy in the progression of diabetic nephropathy.

Renal podocytes are highly specialized, terminally differentiated and nonproliferative cells that exhibit high autophagic activity under non-stress conditions (81). Alterations in autophagy have been observed in diabetic podocytes (82, 83), indicating that regulating autophagy to maintain homeostasis could be a potential target for treating diabetic nephropathy. In a study by Olivia Lenoir et al. (84), renal podocytes of mice were exposed to high sugar levels for 48 hours, which induced autophagy. After being exposed to high glucose levels for 15 days, autophagy was inhibited. *In vivo* experiments on mice induced with STZ to develop T1DM showed that autophagy in renal podocytes was induced at 4 weeks, but became inhibited and resulted in renal podocyte damage at 8 weeks. Knocking down the Atg5 gene in renal podocytes accelerated diabetes-induced deterioration, leading to glomerular filtration barrier leakage and glomerulosclerosis. In diabetic conditions, the expression of Beclin-1, Atg12-5 and LC-3 in renal podocytes was found to be inhibited both *in vitro* and *in vivo*, leading to damage in renal podocytes due to the inhibition of autophagy (85). In a high-fat diet (HFD)-induced diabetes model, podocyte-specific autophagy-deficient mice showed a loss of renal podocytes and massive proteinuria (86). Markus Gödel et al. (87) discovered that mTORC1 was highly activated and autophagy was inhibited in renal podocytes of T2DM mice and patients. However, when mTORC1 gene copies were reduced during the experiment, the disease process in diabetic nephropathy was significantly improved. Wei et al. (88) observed that in a high glucose state, autophagy levels were reduced in human renal podocytes. However, when treated with the autophagy activator rapamycin, autophagy was activated and insulin response was enhanced. This suggests that autophagy regulates insulin responsiveness and that activation of autophagy can improve cellular damage in human podocytes. The use of the mTORC1 inhibitor rapamycin has shown significant improvement in the progression of diabetic nephropathy in T2D rats (89). Further studies by Xiao et al. (90) on STZ-induced T1DM mice have also shown that rapamycin treatment increases autophagosomes and attenuates renal podocyte fusion, indicating that rapamycin improves renal injury in diabetic mice by increasing autophagic activity and inhibiting podocyte apoptosis. However, it is important to note that AGEs inhibit autophagosome formation and renewal in renal podocytes by activating mTOR and inhibiting nuclear translocation of the pro-autophagic TFEB, ultimately causing renal podocyte injury (91). These experimental results suggest that long-term high glucose levels reduce the autophagy level of renal podocytes, leading to renal podocyte damage and the eventual development of diabetic nephropathy.

In addition to renal podocytes, proximal tubular epithelial cells also have a significant role in maintaining renal function. During the course of diabetes, autophagy in proximal tubular epithelial cells undergoes significant changes (82, 92–94). In 1992, Barbosa et al. discovered that in STZ-induced rats, the volume and density of autophagic vesicles in proximal tubular cells were significantly reduced, which led to the accumulation of autophagic substrates (95). When the autophagy-associated gene 7 (Atg7) in the proximal tubular cells was removed, diabetic mice experienced defective autophagy, resulting in more severe renal hypertrophy, tubular damage, inflammation, fibrosis, and proteinuria (96). Kosuke Yamahara et al. (97) discovered that kidney biopsy specimens from type II diabetic patients exhibited deficient autophagy. Additionally, high glucose and obesity were found to significantly inhibit the protective role of autophagy in proximal renal tubular cells. The overactivation of mTORC1 resulted in defective autophagy, which induced proximal tubular cell injury. However, when the diet of diabetic animals was restricted or altered, autophagy was significantly restored, leading to an improvement in diabetic nephropathy (98–100).

Some drug, such as *metformin*, has been found to reduce renal injury in diabetic rats by regulating autophagy through the Sirt1/FoxO1 signaling pathway (101, 102). *Prostaglandin E1 (PGE1)* has also been shown to restore autophagy and insulin resistance in the kidney of type II diabetic (T2DM) rats and promote autophagy-related fibroblast growth factor 21 (FGF21) protein expression, thereby reducing insulin resistance (103). In addition, experimental data from liraglutide treated Male 8-week-old spontaneously diabetic Torii (SDT) fatty rats showed increased expression of phosphorylated (p)-eNOS and p-AMPK in glomeruli, downregulated expression of p-mTOR, and increased expression of LC3B-II, indicating that liraglutide plays a protective role in the kidney by activating autophagy (104). The sodium-dependent glucose transporters 2 (SGLT2) located in the proximal tubule are responsible for reabsorbing 90% of the glucose that is filtered through the glomerulus (105). Studies have shown that SGLT2 inhibitors induce AMPK and SIRT1 to stimulate autophagy, which helps to alleviate cellular stress, as well as glomerular and tubular injury (106). In a study involving 8-week-old male db/db mice, administration of SGLT2 inhibitor Empagliflozin and DPP4 inhibitor Linagliptin resulted in enhanced autophagy in renal podocytes, indicating that both drugs can attenuate the progression of diabetic nephropathy through the autophagic pathway. This resulted in the attenuation of thylakoid expansion, podocyte foot process loss, and urinary albumin excretion (107). Recent studies have demonstrated that *Empagliflozin* can enhance autophagic activity in renal tubular cells exposed to high glucose levels, thereby providing a protective effect on the kidneys (108). Additionally, *GLP-1*, a crucial pro-intestinal hormone, has been found to stimulate autophagy through the AMPK/mTOR signaling pathway and related proteins, ultimately alleviating diabetic nephropathy (109).

**Figure 7:**
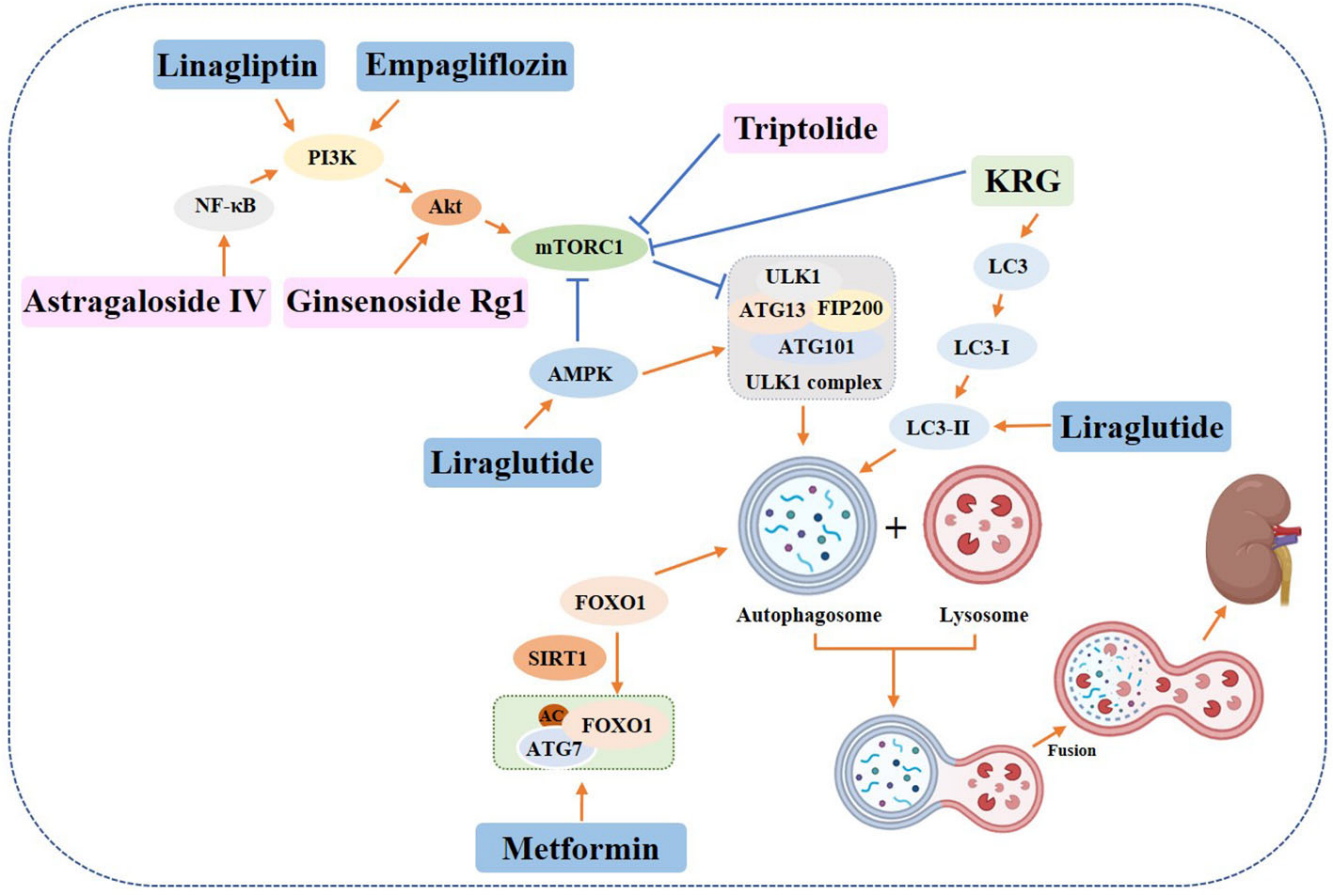
Role of autophagy in diabetic nephropathy. Yellow rectangle: Traditional Chinese compounds; Blue rectangle: Chemical drugs; Pink rectangle: Monomers from Chinese Herbal; Gray rectangle: Experimental Chemicals. →: activate: ⟞: inhibit.

Botanicals have been found to have a protective effect on the kidneys in diabetic individuals by inducing autophagy (110, 111). A study conducted on STZ-induced diabetic rats showed that *Korean red ginseng* treatment resulted in an upregulation of LC3 and a downregulation of p62 in rat kidney cells. Additionally, there was an increase in the levels of ATG7 and inhibition of mTOR, which suggests the activation of autophagy and subsequent recovery from kidney injury (110). The effectiveness of *resveratrol* in reducing high glucose-induced apoptosis was investigated in db/db mice and renal podocytes (112). The activation of autophagy involving miR-383-5p was found to be the mechanism behind this attenuation. Additionally, *triptolide* was found to restore autophagy through the miR-141-3p/PTEN/Akt/mTOR pathway, which resulted in the attenuation of fibrosis (113). According to research, *Astragaloside IV (AS-IV)* has the ability to induce autophagy through the AMPKα pathway (114) or SIRT1 deacetylation of NF-κB p65 subunit (115), which can help alleviate the symptoms of diabetic nephropathy. Another study found that *Ginsenoside Rg1* can attenuate podocyte EMT by enhancing AKT/GSK3β/β-linked protein pathway-mediated autophagy, indicating its potential therapeutic benefits for DN (116) (**Figure 7**).

The results indicate that enhancing autophagic activity could be a promising approach to treating diabetic nephropathy. Nevertheless, some studies suggest that reduced autophagy may also have a protective effect on the kidneys against T2DM damage (117). Further experiments are needed to confirm the exact role of autophagy in this context.

### 3.3 Autophagy and diabetic hepatopathy

The liver is an important organ involved in glucose regulation, lipid metabolism, and insulin action. NAFLD is a widespread liver disease (118), and its disease progression is closely related to T2DM as well as IR (119). As liver disease progresses, IR is exacerbated, and IR is considered a critical event affecting T2DM and NAFLD (120). Global epidemiological statistics for 2019 (121) showed that 55.5% of patients with T2DM worldwide develop NAFLD. As early as 2016, the European Association for the Study of the Liver, the European Association for the Study of Diabetes, and the European Association for the Study of Obesity strongly recommended: NAFLD screening was performed in patients with established T2DM and T2DM screening was performed in patients with NAFLD (122). These two pathological states of T2DM and NAFLD seem to be difficult to say who is the cause and who is the effect, but they share similar complex pathophysiological mechanisms, such as insulin resistance, chronic hyperglycemia, lipotoxicity, low-grade inflammation and increased oxidative stress, and both act synergistically to increase adverse clinical outcomes (123, 124). Diabetics are two-fold more likely to develop NAFLD and vice versa (125). Moreover, T2DM accelerates the progression of NAFLD, allowing NAFLD to rapidly progress to nonalcoholic steatohepatitis (NASH), cirrhosis, and hepatocellular carcinoma (126). In 2020, international experts from 31 organizations proposed the definition of MAFLD (Metabolic dysfunction-associated fatty liver disease) to replace NAFLD, where one of the diagnostic criteria is: confirmed T2DM (127). Although the change in terminology from NAFLD to MAFLD is still under intense debate, it largely demonstrates the close relationship between liver disease and T2DM and the underlying metabolic dysfunction. Studies have shown that autophagy is closely related to NAFLD during its development (128). Impaired autophagic flux was observed in the liver of NAFLD patients, NAFLD mouse models and lipid-overloaded human hepatocytes (129). Autophagy is closely related to hepatic lipid metabolism, and inhibition of autophagy increases triglyceride and lipid droplets *in vitro* and *in vivo*, promoting lipid accumulation, which further inhibits autophagy and increases lipid retention (130). Chloroquine (an autophagy inhibitor) exacerbates hepatic steatosis and liver injury, while carbamazepine and rapamycin (2 autophagy activators) enhance ethanol-induced macroautophagy in hepatocytes *in vitro* and *in vivo* (131). Inhibition of the autophagy-associated gene Atg7 *in vitro* and *in vivo* resulted in defective insulin signaling and elevated endoplasmic reticulum stress. In contrast, restoration of Atg7 expression in the liver resulted in reduced endoplasmic reticulum stress, enhanced hepatic insulin action, and increased systemic glucose tolerance in obese mice (132). Branched-chain amino acids (BCAAs, including leucine, isoleucine, and valine), which are abundant in high-protein foods, are thought to be closely associated with the NALFD-related metabolic disease T2DM. Excess circulating BCAAs activate mTOR and inhibit autophagy, and the blockage of autophagy hinders the self-repair mechanism of hepatic lipotoxicity and increases apoptosis (133). Most of the drugs currently used to treat diabetic liver injury are single hypoglycemic and hepatoprotective drugs, which are ineffective and have significant side effects.

Some chemical drugs can alleviate the symptoms of diabetic liver disease by regulating autophagy. Studies have shown that *Empagliflozin*, an SGLT-2 inhibitor, in addition to its significant hypoglycemic effect, can also enhance autophagy in liver macrophages via the AMPK/mTOR signaling pathway and further inhibit the IL-17/IL-23 axis-mediated inflammatory response, thereby significantly ameliorating liver injury in mice with T2DM combined with NAFLD (134). It has also been shown that Empagliflozin can improve hepatic steatosis through the AMPK-TET2 autophagic pathway (135). *Liraglutide* promotes autophagy through the AMPK/mTOR pathway to reduce lipid accumulation and exert anti-lipotoxic effects (136, 137). Vitamin D_3_ may improve hepatic lipid abnormalities by activating the autophagy-regulated AMPK/Akt-mTOR signaling pathway in diabetics (138). A report on the active form of vitamin D, *1,25(OH)_2_D_3_*, suggests that it can prevent hepatic lipid degradation by stimulating ATG16L1-mediated autophagy (139).

**Figure 8:**
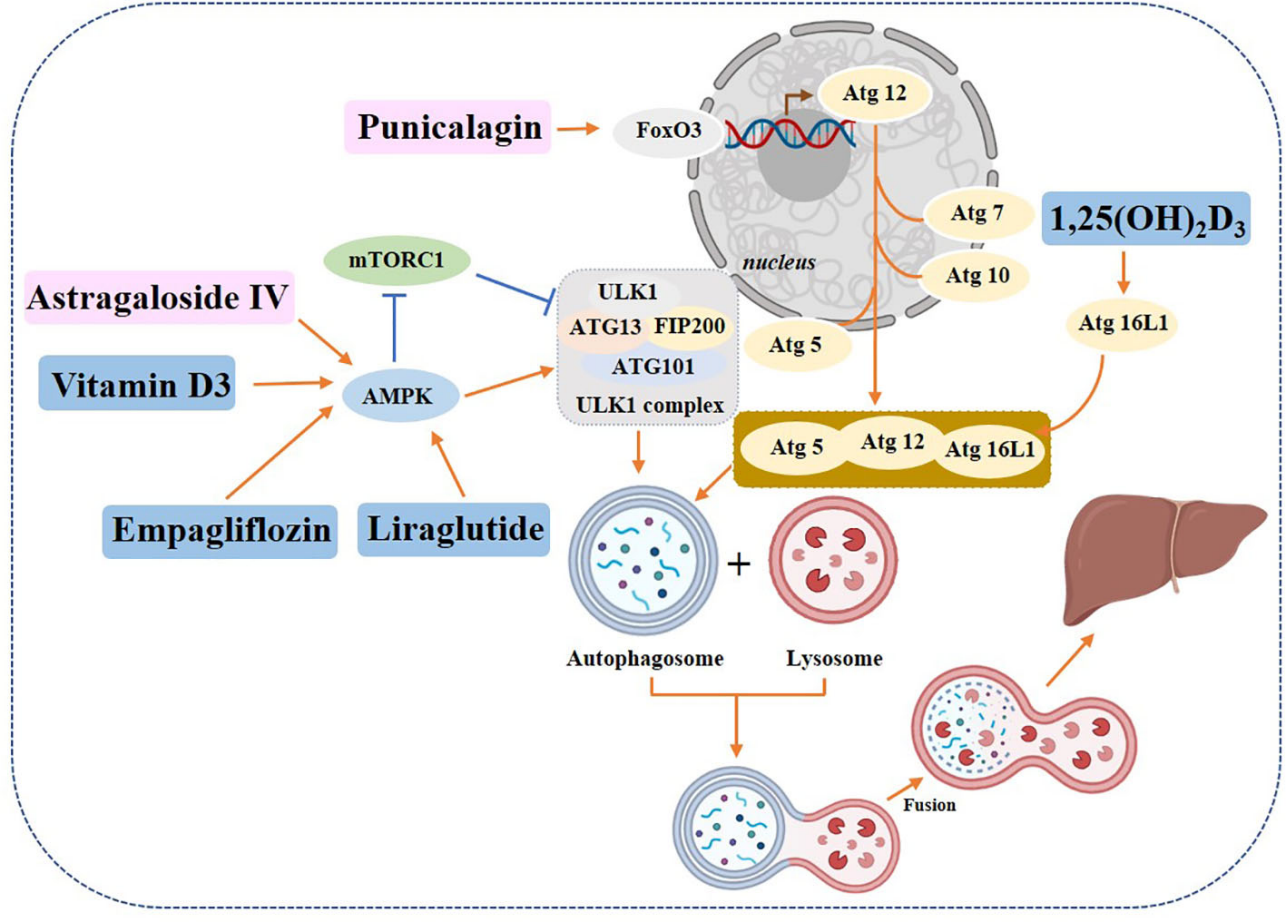
Role of autophagy in diabetic hepatopathy. Yellow rectangle: Traditional Chinese compounds; Blue rectangle: Chemical drugs; Pink rectangle: Monomers from Chinese Herbal; Gray rectangle: Experimental Chemicals. →: activate; ⟞: inhibit.

Plant-derived monomeric compounds also protect against liver injury caused by T2DM. Punicalagin (PU) administration to C57BL/6J mice (at a dose of 20 mg/kg/day) and HepG2 cells (at doses of 5, 10, and 20 μM) significantly improved liver histology, reversed serum biochemical abnormalities and increased the number of autophagosomes in the liver of T2DM mice. The experimental results showed that PU restored autophagy through the Akt/FoxO3a signaling pathway, thereby protecting against T2DM-induced liver injury (140). Astragaloside alleviated liver injury in type 2 diabetes by promoting AMPK/mTOR-mediated autophagy (141) (**Figure 8**).

Taken together, therapies that restore autophagic flux may attenuate or prevent the progression of T2DM liver disease.

### 3.4 Autophagy and diabetic cardiomyopathy

Diabetic Cardiomyopathy is divided into two main categories: one affecting the large vessels (large arteries, including the aorta, femoral arteries and coronary arteries) and the other affecting the microvasculature (small vessels, including capillaries of the eyes, kidneys and nerves) (142). People with diabetes have a 3 to 10-fold increased risk of cardiovascular complications compared with those with normal blood glucose (143). And cardiovascular complications of diabetes increase the risk of myocardial infarction, stroke, and limb loss. The lethality of cardiovascular complications of diabetes is extremely high and is the leading cause of death (144). The myocardium is particularly vulnerable to the negative effects of the hyperglycemic state because it is both an insulin-sensitive and glycolysis-dependent tissue (145). Persistent hyperglycemia inhibits cardiomyocyte autophagy (146, 147). Studies have shown that abnormal cellular metabolism and accumulation of damaged/defective organelles have been observed in cardiomyocytes with diabetic cardiomyopathy (148) and abnormal regulation of autophagy has been observed in preclinical trials in the diabetic heart (149). There is growing evidence that autophagy plays an important role in cardiovascular disease (150–153), restoration of autophagy is cardioprotective and impaired autophagy leads to cardiac damage (154, 155). In research on diabetic cardiomyopathy, modulation of autophagy (whether it is enhanced or diminished needs to be confirmed) improved cardiac function in T2DM rats (156). SGLT2 inhibitors reduced the incidence of cardiovascular death and hospitalization for heart failure by activating autophagy (157). However, it has also been shown that the inhibition of autophagy in cardiomyocytes by sustained hyperglycemia is an adaptively protective mechanism that limits the cytotoxicity of high glucose. When cardiomyocytes in a high-glucose state were treated with an autophagy inhibitor (3-MA) or silencing of autophagy-associated genes (Atg7), autophagy was inhibited and high glucose-induced cardiomyocyte death was attenuated. Contrary to expectations, increasing autophagy through the use of autophagy activators such as rapamycin or by overexpressing Beclin-1 or Atg7 actually made cardiomyocytes more susceptible to the toxic effects of hyperglycemia (146). There are also studies with the same findings that suggest that insulin resistance overactivated autophagy in cardiomyocytes, thus preventing the survival of cardiac cells in diabetics. When autophagy in diabetic cardiomyocytes is inhibited, the survival rate of cardiomyocytes is raised (145, 158, 159). Feng et al. (160) showed that high glucose levels increased diabetic cardiomyopathy-related factor (DCRF) expression and induced cardiomyocyte autophagy and lowering DCRF expression reduced cardiomyocyte autophagy, reduced myocardial fibrosis, and improved cardiac function in diabetic rats.

Several chemical agents, as well as plant-derived monomers, have been reported to exert therapeutic effects by modulating autophagy. *Zinc*, a protective factor in diabetic cardiomyopathy, may protect the heart by inhibiting autophagy (161). Metformin has also been verified to improve the course of experimental cardiomyopathy by activating autophagy directly or indirectly through the activation of AMPK and SIRT1 (162, 163). Some botanicals can also protect the heart of diabetics by regulating autophagy. *Carnosic acid* reduces diabetic myocardial ischemia-reperfusion injury by enhancing autophagy (164). *Polyphenols from green tea extract* can protect rat hearts by improving autophagy (165). *Quercitrin* has been demonstrated to activate the ERK signaling pathway and enhance cellular autophagy in endothelial progenitor cells in a dose-dependent manner *in vitro* experiments and thus may be used as a therapy for a variety of diseases caused by impaired endothelial function (166). *Resveratrol* enhances autophagy *via* SIRT1/FOXO1/Rab7 axis *in vivo* and *in vitro* and ameliorates myocardial oxidative stress injury in diabetic mice (167). *β-carotene* can inhibit autophagy through activation of PI3K/Akt/mTOR, which in turn exerts cardioprotective effects, and can protect H2c9 cells damaged by advanced glycosylation products (168). *Curcumin* ameliorates diabetic cardiomyopathy by activating AMPK and JNK1, phosphorylated Bcl-2 and Bim and subsequently disrupted their interactions with Beclin1, promoting autophagy and attenuating apoptosis *in vivo* and *in vitro* diabetic models (169).

In addition to diabetic cardiomyopathy, drugs have also been reported to exert therapeutic effects by modulating autophagy in studies related to diabetes combined with atherosclerosis. *α-lipoic Acid sulfate*, a fatty acid present in the human body, protects vascular smooth muscle cells from atherosclerosis in T2DM patients by downregulating autophagy through the AMPK/mTOR pathway (170), but Sirt6-mediated Caveolin acetylation 1 is the key factor in atherosclerosis in T2DM through activation of autophagy (171).

Based on the above findings, in terms of the relationship between autophagy and diabetic macrovascular complications (mainly diabetic cardiomyopathy), some scholars believe that the role of autophagy in diabetic cardiomyopathy is more like the “Goldilocks” phenomenon, i.e. there is a narrow “optimal” autophagic region, and when we adjust autophagy within the “optimal range”, the best therapeutic effect will be achieved (172, 173).

### 3.5 Autophagy and diabetes-associated cognitive decline

Diabetes-associated cognitive decline (DACD) is one of the common complications in diabetics (174). DACD refers to the decline in cognitive function in diabetics, manifested by deficits in learning, memory, reasoning and verbal expression. The long-term hyperglycemia of T2DM significantly increases brain glucose flow (175), producing damage to the central nervous system (176, 177), affecting several cognitive domains and increasing the risk of cognitive dysfunction (178–180). The longer the duration of T2DM, the more likely it is to develop cognitive dysfunction (181). In general, DACD is difficult to reverse. Without effective intervention, diabetes will greatly accelerate the progression from mild cognitive impairment to dementia (182). Here we have to mention that Alzheimer’s disease, a form of cognitive impairment, has overlapping molecular and biochemical features with T2DM (183), hence Alzheimer’s disease is also called type III diabetes (184). In recent years, an increasing number of scientific studies have confirmed that autophagy plays an important role in the molecular basis of T2DM and cognitive impairment (185, 186). However, findings vary on how autophagy regulates the relationship between diabetes and cognitive impairment. Some findings suggest that T2DM causes enhanced autophagy, which in turn causes deposition of Aβ in the brain and exacerbates cognitive impairment. For example, Son et al. (187) found that insulin resistance promotes Aβ production in the brain by altering insulin signaling and increasing autophagic flux. Ma et al. (188) found that diabetes activates autophagy, but the autophagy-lysosome function is impaired, and the resulting of autophagy-lysosome dysfunction caused Aβ deposition in diabetic cognitive impairment. However, more voices contradict this, and most findings suggest that the development of diabetic cognitive impairment is associated with the reduced autophagic activity. For example, Wu et al. (189) found that reduced autophagic activity of db/db diabetic mice is associated with the accumulation of Aβ, the classic marker of Alzheimer’s disease. Decreased spatial learning, as well as memory capacity in aged diabetic mice, is associated with the inhibition of autophagy marked by the upregulation of p62 and the downregulation of Beclin protein in the hippocampus (190, 191). Excessive activation of the PI3k/Akt/mTOR signaling pathway and inhibition of autophagy can be observed in macrophages of the brains of diabetic animals (192, 193). Up to now, some Chinese traditional herbs’ monomers and compounds, and chemical drugs in the market, can improve diabetic cognitive impairment by modulating autophagy. We’ll cover each in the following.

*Gliflozins*, an oral antidiabetic drug, can restore mTOR to its activated physiological state and prevent the development of neurodegenerative diseases (194). *Metformin* improves cognitive impairment in T2DM by regulating autophagy through an AMPK-dependent pathway (195). A high dose of *liraglutide* (200 μg/kg) alleviated learning and cognitive impairment in T2DM rats enhanced autophagic signaling and improved cognitive function by activating PI3K/Akt and AMPK pathways to inhibit p-mTOR expression (196). *Exendin-4* activated PKA and PI3K/Akt signaling pathways in rat cerebral cortex to promote autophagy and inhibit the onset of apoptosis (197). *Melatonin* activates autophagy through the TLR4/Akt/mTOR pathway and improves learning and memory in type II diabetic mice (198).

**Figure 9:**
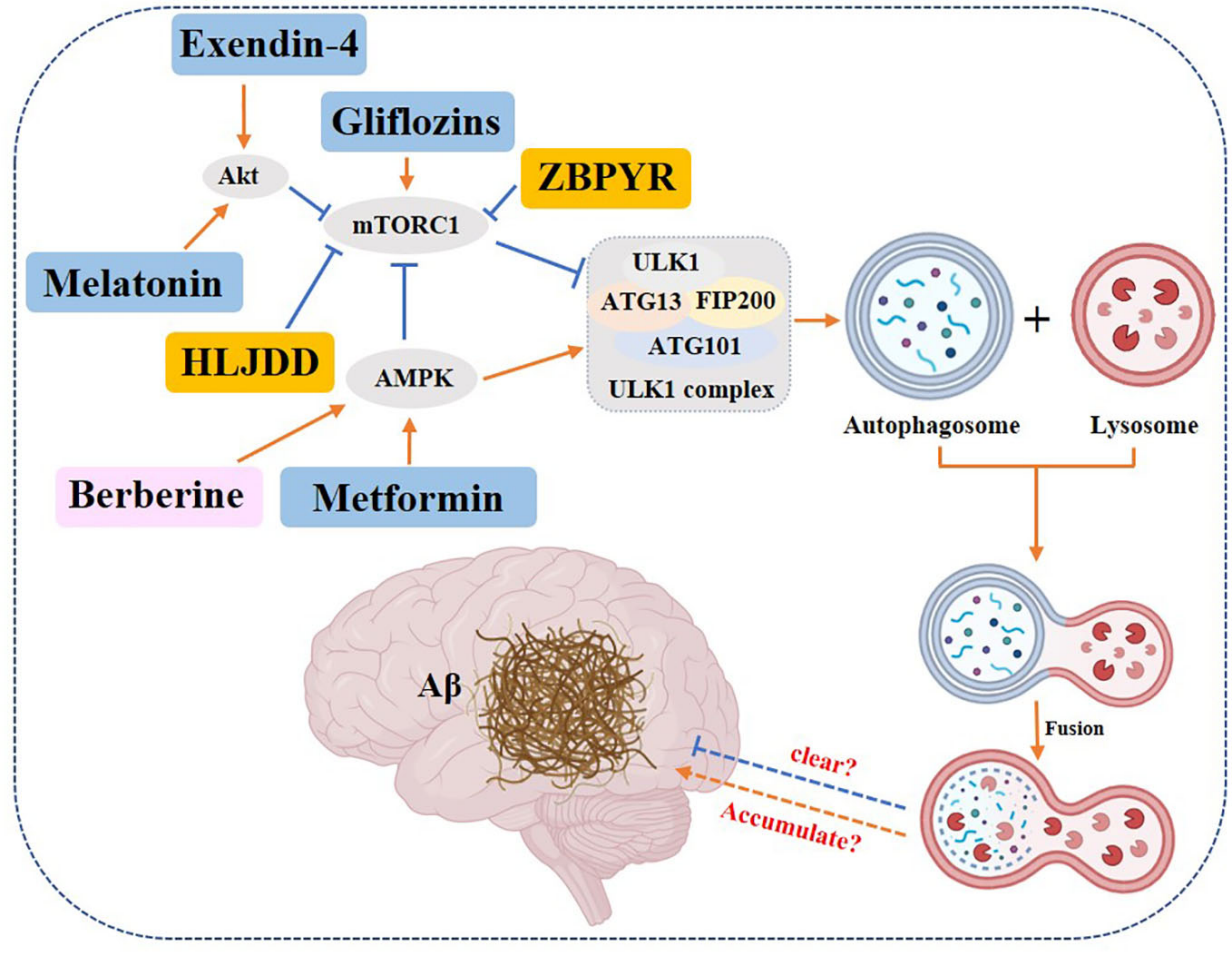
Role of autophagy in DACD. Yellow rectangle: Traditional Chinese compounds; Blue rectangle: Chemical drugs; Pink rectangle: Monomers from Chinese Herbal; Gray rectangle: Experimental Chemicals. → activate; ⟞: inhibit.

In addition to chemical drugs, there are also reports of Chinese herbal monomers and compounds targeting autophagy to improve cognitive function in diabetics. *Huanglian Jiedu Decoction* can inhibit the activation of inflammasome NLRP and upregulate autophagy (199). ZDF rats administered with *ZiBuPiYin Recipe* were found to obtain improvement in learning memory impairment, presumably achieved by inhibiting mTOR upregulation, enhancing autophagy, and promoting Aβ clearance (200). Besides traditional Chinese compounds, the traditional Chinese monomer was reported to have the same biological activities. *Berberine*, a quaternary alkaloid isolated from Coptis, a traditional Chinese medicine, enhances cellular autophagy and attenuates neuronal apoptosis by activating the AMPK/mTOR pathway (201) (**Figure 9**).

In summary, most of the studies concluded that enhanced autophagy can promote the clearance of Aβ and thus improve the condition of DACD.

### 3.6 Autophagy and diabetic retinopathy

Diabetic retinopathy (DR) is a chronic and progressive complication of type 2 diabetes mellitus (T2DM), primarily resulting from prolonged hyperglycemia (202). It is also the leading cause of blindness in diabetic patients. The incidence of DR has significantly increased due to the elongation of human lifespan and the rising prevalence of T2DM. According to WHO statistics (203), it is projected that approximately one-third of diabetic patients worldwide will develop diabetic retinopathy by 2025. Recent scientific research has revealed that diabetic retinopathy is not only a vascular condition but also a neurodegenerative disease (204). Autophagy has been found to have a significant impact on the development and treatment of diabetic retinopathy (205–207). For instance, The dysregulation of mTOR-dependent autophagy is a contributing factor to the loss of retinal ganglion cells in streptozotocin-induced diabetic retinopathy. This loss further worsens the condition of diabetic retinopathy (208). Autophagy-related proteins are highly expressed in the retinas of both normal individuals and diabetic patients (209, 210). Notably, the Atg16L1 gene shows a significant up-regulation, indicating its involvement in the cell death process associated with diabetic retinopathy (211). An experiment was conducted to observe the effects of high glucose conditions on human retinal pigment epithelial cells (ARPE-19). The cells were exposed to 30 mmol/L D-glucose and subsequently observed. Subsequently, the observed results indicated a reduction in ARPE-19 cell viability, an increase in apoptosis, and elevated protein expression of Bax, Caspase-3, and LC3-II/LC3-I. Conversely, the expression of Bcl-2, p62, and p-mTOR was decreased. Notably, the activation of autophagy was observed. Moreover, the effects induced by high glucose were reversed upon treatment with the autophagy inhibitor 3-MA (212). The same results were obtained in another experiment. The expression of LC3-I in ARPE-19 cells was significantly reduced under high glucose stress. Additionally, intracellular ROS levels were significantly elevated, and the number of autophagosomes was significantly increased. These findings indicate that high glucose activates autophagy (213). Contrary experimental results have also been reported regarding the regulation of autophagy in response to high glucose. When mouse retinal explants were treated with high glucose (HG) for 10 days, it was observed that there was a significant increase in explant apoptosis, while autophagic flux showed a significant decrease. The findings indicate a potential relationship between autophagy dysfunction and the mechanism of DR. There have been reports of drugs targeting autophagy for the treatment of DR, which are described below.

Treatment of retinal explants treated with the growth hormone analog *octreotide (OCT)* demonstrated that the explants were shielded from apoptosis and exhibited enhanced autophagic activity. Furthermore, the addition of the autophagy inhibitor chloroquine completely negated the anti-apoptotic effect exerted by OCT (214). The study on *Glucagon-like peptide-1 (GLP-1)* treatment in rats with type II diabetes demonstrates that GLP-1 reduces oxidative stress-induced autophagy by activating the GLP-1R-ERK1/2-HDAC6 signaling pathway. This mechanism ultimately leads to an improvement in diabetic retinopathy (215). *PG545*, a heparanase inhibitor, was administered to diabetic mice via intraperitoneal injection at a dose of 20 mg/kg/d. This treatment effectively slowed down the diabetes-induced changes in body weight and reduced fasting blood glucose levels in mice. Additionally, PG545 was observed to mitigate the reduction in retinal thickness and the formation of microaneurysms both *in vivo* and *in vitro*. These beneficial effects were attributed to the promotion of autophagy and the inhibition of inflammatory responses by PG545 (216). *β-hydroxybutyrate (BHB)* has been previously employed as a trophic agent for brain-derived neurotrophic factor (BDNF). In experimental studies, the administration of BHB (25-50-100 mg/kg) to treat STZ-induced C57BL/6J diabetic mice resulted in a noteworthy reduction in autophagy activation in M1 microglia, while simultaneously increasing retinal BDNF levels (217). *Arjunolic acid*, an active ingredient derived from Cyclocarya paliurus, was investigated in a rat model of STZ-induced diabetes mellitus. The experimental animals were orally administered doses of 10 mg/kg and 30 mg/kg of Arjunolic acid for a duration of 10 weeks. The study revealed that Arjunolic acid effectively safeguards retinal cells against oxidative stress and inflammation induced by STZ. This protection is mediated through the autophagy pathway, which is regulated by AMPK/mTOR/HO-1 (218). *Artesunate (ART)*, an active ingredient derived from the traditional Chinese medicine *Artemisia annua* L., was intravenously injected into STZ-induced diabetic rats. The study demonstrated that ART improved the physiological state of the retina in a positive manner. The findings indicated that the expression of Beclin-1 and the ratio of LC3II/I were up-regulated, while p62 was down-regulated. Additionally, the AMPK/SIRT1 pathway was activated, suggesting that ART can activate autophagy and potentially play a therapeutic role (219). Similarly, *quercetin* treatment of human retinal microvascular endothelial cells (HRMECs) cultured *in vitro* at high glucose levels showed a dose-dependent inhibition of autophagy (220). On the other hand, *Gypenoside XVII (Gyp-17)*, a natural product isolated from the traditional Chinese medicine Panax ginseng, was used for the treatment of early diabetic retinopathy (DR) in db/db mice and Müller cells. The results demonstrated that Gyp-17 significantly increased the expression of pro-autophagy-related proteins and could prevent early DR by enhancing autophagy (221). *Norkurarinone* and *isoxanthohumol* have been shown to enhance cellular oxidative stress, activate the PI3K/AKT/mTOR signaling pathway, and regulate autophagy dysregulation. These effects have been found to protect human retinal microvascular endothelial cells under conditions of high glucose and hypoxia (222). *Procyanidin*, a polyphenolic compound, also exhibits anti-diabetic properties. In an experimental study, retinal pigment epithelial cells were initially exposed to high glucose, resulting in reduced cell viability, increased apoptosis rate, and enhanced autophagic flux. However, subsequent administration of procyanidin reversed these changes induced by high sugar levels. Furthermore, when the autophagy agonist rapamycin was reintroduced, retinal pigment epithelial cells exhibited decreased activity and increased apoptosis. This suggests that PC protects retinal pigment epithelial cells by inhibiting autophagy (223). In type II diabetic rat model, it was discovered that *Mingmu Xiaomeng Tablets (MMXM)* administration protected the retina in the diabetic state. This protection was achieved by decreasing the protein expression of LC3-II and p62, reducing the phosphorylation of PI3K, Akt, and mTOR, inhibiting PI3K/Akt/mTOR signaling, and promoting autophagy (224).

This section highlights the dual role of autophagy in DR and its potential as a target for drug therapy. The study reveals that certain drugs can activate autophagy to positively treat DR, while others can inhibit autophagy. However, the exact mechanism of action is still not fully understood and requires further investigation.

## 4 Conclusion

Diabetes mellitus is an endocrine metabolic disease characterized by disorders in glucose and lipid metabolism, leading to increased plasma glucose levels. Its pathophysiology is characterized by a deficiency in insulin secretion, either absolute or relative, with or without increased glucagon activity (15). With accelerated economic development and industrialization, the prevalence of diabetes and the number of diabetics have risen sharply, and diabetes itself and its complications have placed a heavy burden on human health and social development (225, 226). The pathogenesis of diabetes is complex, including both genetic and external environmental factors, and there is an intricate link between diabetes and diabetes complications, and between complications and complications. Autophagy, as a strictly regulated intracellular catabolic process, is closely related to glucose metabolism. One type of autophagy is non-selective autophagy, which refers to autophagy initiated in the starved state. The other type of autophagy is selective autophagy, which removes and recycles harmful or unwanted substances from the cell, such as protein aggregates, damaged mitochondria, accumulated peroxisomes, excess ribosomes, endoplasmic reticulum, and other cellular phases, which, if left to accumulate without timely removal, can lead to human diseases (3). Studies have shown that autophagy is disrupted under a high-calorie diet (51, 88, 97). Impaired autophagy function in pancreatic islet beta cells is associated with beta cell dysfunction and failure. Activation of autophagy improves insulin resistance in the diabetic process, and inhibition of autophagy accelerates the death of islet beta cells. In studies on the relationship between autophagy and diabetic complication, it was found that autophagy is involved in the development of diabetic nephropathy, diabetic hepatopathy, diabetic cardiomyopathy, diabetic cognitive dysfunction, and so on, with different mechanisms of upregulation or downregulation of autophagy, which are briefly summarized here.

### 4.1 Diabetic nephropathy

Both *in vivo* and *in vitro* experiments showed that short-term (48 hours *in vitro*, 4 weeks *in vivo*) high-sugar exposure activated autophagy in renal podocytes, while long-term (15 days *in vitro*, 8 weeks *in vivo*) high-sugar exposure inhibited autophagy (84). And the physiological characteristics of T2DM are precisely the chronic hyperglycemia levels. Experiments have shown that both chronic high blood glucose levels (88), and the accumulation of AGEs (91), as well as the knockout deletion of autophagy-related genes (Atg5-12/Beclin-1/LC-3) (84–86, 96), eventually lead to damage of renal podocytes and proximal tubular epithelial cells by inhibition of autophagy, which in turn causes renal inflammation, fibrosis, proteinuria, and other symptoms of diabetic nephropathy. When the autophagy inhibitor (rapamycin) was used to treat the diabetic nephropathy cells, the autophagic flux was increased and the course of diabetic nephropathy was improved (89, 90). In conclusion, the development of diabetic nephropathy is associated with the inhibition of autophagy, and activation of autophagy can restore the viability of renal podocytes and proximal renal tubular cells, and thus treat diabetic nephropathy.

### 4.2 Diabetic hepatopathy

As an important organ for regulating glucose and lipid metabolism, the progression of liver disease and T2DM are both closely related to metabolic dysfunction. Impaired autophagy has been demonstrated to be present in diabetic liver disease (129). A high protein diet (133), use of autophagy inhibitors (chloroquine) (131), and autophagy gene deletion (Atg7) (132) all lead to disruption of autophagy, which in turn leads to obstruction of hepatic lipotoxic self-repair mechanisms and increases apoptosis, resulting in the development and progression of diabetic liver disease. When autophagy activators are used and expression of the autophagy gene (Atg7) is restored, a positive effect on the treatment of the disease is demonstrated (132). In conclusion, autophagy inhibition plays a negative role in the development and progression of diabetic liver disease, and activation of autophagy may be a new target for the treatment of diabetic liver disease.

### 4.3 Diabetic cardiomyopathy

Cardiac muscle cells are insulin-sensitive tissues and glycolysis-dependent tissues, making them vulnerable to hyperglycemia. Studies have shown that persistent hyperglycemia inhibits autophagy in cardiac muscle cells (146, 147). However, this autophagy inhibition does not always have a negative effect on cardiac muscle cells. When cardiac muscle cells in the high-glucose state were treated with an autophagy inhibitor (3-MA) or silencing of autophagy-related genes (Atg7), autophagy was inhibited and high-glucose-induced cardiac muscle cell death was reduced. Meanwhile, when autophagy is activated with an autophagy activator (rapamycin), the cells are prone to suffer from high glucose toxicity. It has also been shown that insulin resistance over-activates autophagy in cardiomyocytes and prevents cardiomyocyte survival. Therefore, inhibition of autophagy in cardiac muscle cells may enhance their survival rate. The role of autophagy in diabetic cardiomyopathy is considered by some scholars a “Goldilocks” phenomenon, which means that excessive activation or inhibition of autophagy can lead to the development and progression of diabetes and its complications. In order for autophagy to play a positive role, it may be necessary to find an “optimal range” of autophagy, within which autophagy is triggered to obtain the best positive feedback (172, 173).

### 4.4 Diabetic-associated cognitive decline, DACD

Prolonged high sugar levels can produce damage to the central nervous system, affecting cognitive domains and increasing the risk of cognitive dysfunction. The longer the duration of T2DM, the more likely it is that cognitive dysfunction will occur. It has been confirmed that autophagy plays a role in DACD. However, the specific trends of the roles are not the same. Most studies suggest that the occurrence of DACD is related to the inhibition of autophagy, which is suppressed and Aβ accumulates (187). However, some studies have suggested that T2DM activates autophagy, which in turn causes Aβ deposition in the brain. Therefore, we wonder whether the role of autophagy in DACD is similar to that of the “Goldilocks” phenomenon, in which excessive activation or inhibition can lead to accelerated disease progression, and the most appropriate treatment point is within a narrow range.

### 4.5 Diabetic retinopathy

As a vasculopathy and neuropathy, DR is the leading cause of blindness in diabetic patients. Research studies have identified autophagy as a potential target for the treatment of DR. In human retinal pigment epithelial cells, prolonged high glucose levels activate autophagy, while in mice retinal explants, they inhibit autophagy. Therefore, dysregulation of autophagy worsens DR. When considering autophagy as a therapeutic target for DR, certain drugs have shown the ability to prevent or treat DR by activating autophagy and reducing oxidative stress. However, other drugs have beneficial effects by inhibiting autophagy. It is important to note that the role of autophagy in DR is dose-dependent, acting protectively at low doses but promoting cell death at high doses.

Oral hypoglycemic drugs currently used in clinical practice are divided into seven major classes: sulfonylureas, biguanides, alpha-glucosidase inhibitors, thiazolidinediones, glinides, glucagon-like peptide-1 (GLP-1) and its analogs, and DPP-4 inhibitors. Some chemical drugs are mentioned in this review that can treat T2DM and its complications by regulating autophagy. For example, metformin (a biguanide hypoglycemic agent) can exert therapeutic effects on pancreatic β-cell protection (56), diabetic nephropathy (102, 103), cardiomyopathy (163, 164), and DACD (196) by activating autophagy. Liraglutide, a GLP-1 analog, has been shown to enhance autophagy, providing protection to pancreatic β cells (53, 54) and potential treatment for conditions such as diabetic nephropathy and diabetic hepatopathy. However, it is important to note that GLP-1 inhibits autophagy and improves diabetic retinopathy (215). Additionally, Exendin-4 and Linagliptin have been found to activate autophagy, leading to potential benefits in protecting pancreatic β-cells and improving conditions like diabetic nephropathy and DACD (55, 197). Empagliflozin (SGLT2 inhibitor) can be used to treat diabetic nephropathy and diabetic hepatopathy by enhancing autophagy (107, 108, 134, 135), but Gliflozins (SGLT2 inhibitor) is used to alleviate DACD by inhibiting autophagy (194). In addition to the commonly used glucose-lowering drugs in clinical practice, there are alprostadil (prostaglandins), vitamin D3 and 1,25(OH)_2_D_3_ (Vitamin), α-lipoic acid (a fatty acid in the human body), melatonin (Indole heterocycles), zinc, octreotide (growth hormone), PG545 (heparanase inhibitor), β-hydroxybutyrate have also been shown in experiments to treat diabetes-related complications by modulating autophagy (138, 139, 161, 170, 214, 216, 217). Among them, zinc, α-lipoic acid, GLP-1 and β-hydroxybutyrat alleviate the symptoms of diabetic cardiovascular complications by inhibiting autophagy, while the rest of the drugs play a therapeutic role in treating the disease by upregulating autophagy.

Most chemical drugs are single drug components, and some of them can produce unexpected adverse effects. For example, mTOR inhibitors can activate autophagy to provide kidney protection, but at the same time, they can inhibit cell growth and hinder kidney repair. Considering that diabetes and diabetic complications, as well as diabetic complications and complications, do not exist independently but are complexly connected and closely linked, it is promising to consider the application of herbal medicine for the treatment of metabolic diseases. In traditional Chinese medicine formula, Yunpi Heluo Decoction can activate autophagy by regulating the SIRT1-FoxO1 signaling pathway in skeletal muscle, while improving lipid metabolism, attenuating insulin resistance and exerting a protective effect on pancreatic β cells (58). Huanglian Jiedu Decoction can improve DACD by inhibiting the activation of NLRP3 inflammatory vesicles upregulating autophagy (199). Zibu Piyin Recipe improves DACD by inhibiting mTOR upregulation, enhancing autophagy, and promoting Aβ clearance (200). Xiaokeping induces mTOR-mediated autophagy and reduces apoptosis in pancreatic β-cells, with a potential protective effect on pancreatic β-cells in high glucose toxicity (59). Mingmu Xiaomeng Tablets exerts a protective effect on the retina during diabetic states by inhibiting the PI3K/Akt/mTOR signaling pathway and promoting autophagy (224). Besides the TCM formula, herbal extracts have also shown good efficacy in animal and cellular models. For example, Morus alba leaves ethanol extract could protect islet cells by inducing AMPK/mTOR-mediated autophagy and by upregulating LC3-activated autophagy, respectively (60). Ginseng extract could treat diabetic nephropathy by upregulating LC3 and downregulating p62 (110). Green tea extract polyphenols could also treat diabetic cardiomyopathy in rats by improving autophagy (165). Monomers from TCM, such as kaempferol and silymarin can maintain cellular energy homeostasis, increase autophagy, and exert insulin β-cell protection through AMPK/mTOR pathway (62), activating autophagy-dependent ERs (63) respectively. Resveratrol, triptolide, astragaloside IV, ginsenoside Rg1, and ferulic acid can exert their effects in the treatment of diabetic nephropathy through activation of miR-383-5p (112), ERK signaling, miR-141-3p/PTEN/Akt/mTOR pathway (113), and AKT/GSK3β/β-linked protein pathway-mediated autophagy (116), respectively. Among them, astragaloside IV can improve diabetic hepatopathy by promoting AMPK/mTOR-mediated autophagy (114, 115), and PU can restore autophagy through Akt/FoxO3a signaling pathway to treat diabetic hepatopathy (140). Resveratrol, carnosic acid, beta carotene, and curcumin can activate autophagy through the SIRT1/FOXO1/Rab7 axis (167), PI3K/Akt/mTOR (168), activate AMPK and JNK1, phosphorylated Bcl-2 and Bim and subsequently disrupted their interactions with Beclin1 to treat diabetic cardiomyopathy (169). Quercetin can activate the ERK signaling pathway in a dose-dependent manner and enhance cellular autophagy in endothelial progenitor cells (166). Berberine enhances cellular autophagy, attenuates apoptosis and improves DACD by activating the AMPK/mTOR pathway (201). Arjunolic acid, artesunate, quercetin, gypenoside XVII, norkurarinone and isoxanthohumol, procyanidin treat diabetic retinopathy through activating or inhibiting autophagy (218–223). All the above-mentioned drugs have been summarized in **Supplementary Table 1**.

In the mechanistic exploration of complex metabolic diseases T2DM as well as drug development, as a mechanism to regulate energy metabolic homeostasis autophagy has now been shown to play a role in the treatment of diabetes. Targeting the regulation of autophagy is likely to have great advantages for the treatment of T2DM and its complications. It is however important to note that autophagy is inherently two-sided, and its two-sidedness is even more evident in its treatment of diabetes and its complications. Therefore, elucidating the range within which autophagy can be regulated for optimal therapeutic effect is the focus of future research.

## Author contributions

XZ drafted this manuscript. L-YB made additions to the text and made changes for contextualization. D-RP drew the structural formula for the compounds. XL and L-FY consulted references. D-DC and YRW organized the references. YG reviewed and revised the manuscript. All authors contributed to the article and approved the submitted version.
