# Supplementary material for: The role of autophagy in the treatment of type II diabetes and its complications: a review
Source: Front Endocrinol (Lausanne). 2023 Sep 21;14:1228045. doi: 10.3389/fendo.2023.1228045 (PMC10551182; doi:10.3389/fendo.2023.1228045)
Supplement: Supplementary file 1 [file Table_1.docx]

|  |  |  |  |  |  |  |
| --- | --- | --- | --- | --- | --- | --- |
|  | **Therapies** | **Diseases** | **Mechanisms** | **Experimental Models** | **Autophagy regulation** | **Refs** |
| **Chemical Drugs** | **Liraglutide** | Pancreatic β cells | p-FoxO1↑ | INS-1 cells | up | (54) |
|  |  | DN | p-AMPK ↑  p-mTOR ↓  LC3B-II ↑ | Male 8-week-old spontaneously diabetic Torii (SDT) fatty rats | up | (105) |
|  |  | DH | AMPK ↑  mTOR ↓ | NALFD rat model and HepG2 cells (138);  male 8-week-old C57BL/6 mice and human hepatic L-O2 cell (139) | up | (137, 138) |
|  |  | DACD | p-AMPK ↑  PI3K ↑  p-Akt ↑  p-mTOR ↓ | 30 male Goto-Kakizaki (GK) rats (age, 32 weeks; weight, 300-350 g) and 10 male Wistar rats (age, 32 weeks; weight, 300-350 g) | up | (197) |
|  | **Exendin-4** | Pancreatic β cells | autophagosome clearance ↑ | Eight-week-old male Sprague Dawley rats/ INS-1 cells | up | (56) |
|  |  | DACD | p-mTOR ↓  Beclin-1 ↑ | 8-month-old (middle-aged)  male Wistar control and T2D GK rat (a non-obese model that spontaneously develop T2D early in life) | up | (198) |
|  | **Metformin**  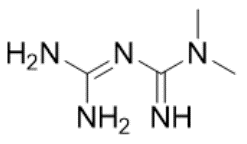 | Pancreatic β cells | p-AMPK ↑ | Mouse insulinoma (MIN6) cells | up | (57) |
|  |  | DN | Sirt1/FoxO1 mediated autophagy ↑ | Eight weeks old male Sprague-Dawley rats with a well-established high-fat diet and low-dose streptozotocin, rat mesangial cells (RMCs) (103);  Male Sprague–Dawley (SD) rats (210 ± 10 g, 6 weeks old) with high-fat feed (104) | up | (102, 103) |
|  |  | DC | p-AMPK ↑ | OVE26 mice | up | (163, 164) |
|  |  | DACD | P62 ↓  LC3II/I ↑  Beclin-1 ↑ | Mouse hippocampal neuron cells HT22, six-week-old male db/db (BKS.Cg-Dock7m+/+Leprdb/Nju) mice | up | (196) |
|  | **Prostaglandin E1，PGE1**  **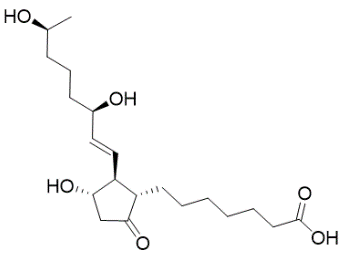** | DN | P62 ↓  LC3II/I ↑  Beclin-1 ↑ | HK-2 cell lines,  Adult male Sprague-Dawley rats (weighing 130–150 g) were fed a high-fat diet (HFD) and a low dose of STZ to develop a rat model of type2 diabetes | up | (104) |
|  | Empagliflozin  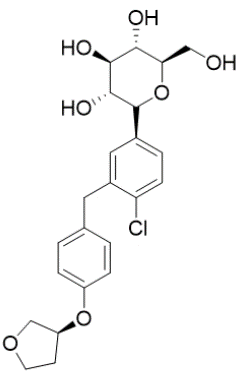 | DN | glomerular autophagy ↑ | db/db-specific pathogen-free mice (BKS.Cg-Dock7m+/+Leprdb/J, stock #000642) | up | (108) |
|  |  |  | mTOR ↓ | eight-week-old male C57/BL6J mice by intraperitoneal injection of streptozotoxin at a dose of 50 mg/kg for 5 consecutive days | up | (109) |
|  |  | DH | AMPK ↑  mTOR ↓ | Six- to eight-week-old male C57BL/6J mice (20–25 g) | up | (135) |
|  |  |  | AMPK ↑ | Eight-week-old db/db mice (*in vivo* model), HL7702 cells induced with high glucose and palmitic acid (*in vitro* model) | up | (136) |
|  | Linagliptin  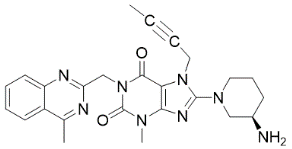 | DN | glomerular autophagy ↑ | db/db-specific pathogen-free mice (BKS.Cg- Dock7m+/+Leprdb/J,  stock #000642) | up | (108) |
|  | Vitamin D3  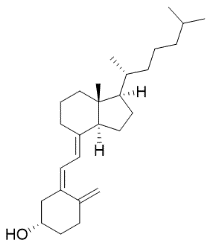 | DH | p-mTOR/mTOR ↓  AMPK ↑  Akt ↑ | Four-week-old male C57BL/6J mice (16–18 g) | up | (139) |
|  | 1,25(OH)_2_D_3_  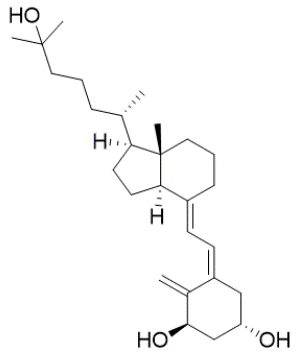 | DH | ATG16L1 ↑ | C57BL/6 male mice (18-19 g), HepG2 cells | up | (140) |
|  | Zinc | DC | LC3B ↓ | Male Wistar rats (180-220 g) induced by high-fat diet and STZ administration | down | (162) |
|  | Vitamin B_6_  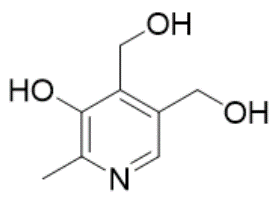 | Islet β cytoprotective effect | mTOR ↓  LC3-II/LC3-I ↑ | RIN-m5F cells | up | (58) |
|  | Alpha-lipoic acid  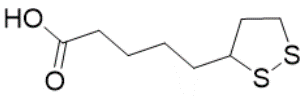 | atherosclerosis | AMPK ↓  mTOR ↑ | 101 adults (aged ≥18 years) with a diagnosis of T2DM from August 2016 to December 2016 | down | (171) |
|  | Gliflozins  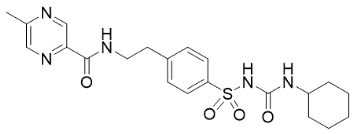 | DACD | mTOR ↑ | / | down | (195) |
|  | Melatonin  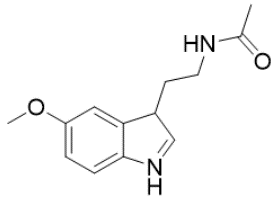 | DACD | Akt ↑  mTOR ↓ | 8-week-old male C57BL/6J mice | up | (199) |
|  | Octreotide | DR | mTOR ↓ | Ex vivo mouse retinal explants | up | (215) |
|  | Glucagon-like peptide-1 | DR | GLP-1R-ERK1/2-HDAC6 signaling pathway ↑ | retinal cells in type 2 diabetic rats | down | (216) |
|  | PG545 | DR | Beclin1 ↑  p62 ↑ | Male 6-week-old C57BL/6J  mice | up | (217) |
|  | β-hydroxybutyrate | DR | LC3B ↓ | C57BL/6J mice | down | (218) |
|  | Arjunolic acid | DR | AMPK ↑  LC3II/I ↑  p62 ↓  mTOR ↓ | STZ-induced diabetic model of rats | up | (219) |
|  | Artesunate | DR | Beclin-1 ↑  LC3II/I ↑  P62 ↓ | Rats with streptozotocin-induced DR | up | (220) |
| **Chinese Herbal Compound** | Yunpi Heluo decoction (YPHLD) | Islet β cytoprotective effect | SIRT1-FoxO1 mediated autophagy ↑ | male ZDF rats (SPF) aged 8 weeks and weighing 240–250 g, male ZL rats aged 8 weeks and weighing 180–200 g | up | (59) |
|  | Huanglian Jiedu Decoction (HLJDD) | DACD | P62 ↓  ATG7 ↑  LC3 ↑ | Sprague-Dawley rats | up | (200) |
|  | ZiBuPiYin Recipe (ZBPYR) | DACD | p-mTOR/mTOR ↓ | Male 6-week-old ZDF (fa/fa) rats | up | (201) |
|  | Xiaokeping  (XKP) | Islet β cytoprotective effect | mTOR ↓  p62 ↓  LC3II/LC3-I ↑ | MIN-6 cells | up | (60) |
|  | Mingmu Xiaomeng Tablets (MMXM) | DR | LC3II ↓  p62 ↓  p-PI3K ↓  p-Akt ↓  p-mTOR ↓ | male Sprague Dawley rats (6 to 8 weeks old) | up | (225) |
| **Chinese Herb Extracts** | Morus alba leaves ethanol extract | Islet β cytoprotective effect | LC3II/LC3-I ↑  p-mTOR ↓  p62 ↓  p-AMPK ↑ | Male Sprague-Dawley (SD) rats (200 ± 20 g), INS-1 rat insulinoma cells | up | (61) |
|  | Korean red ginseng | DN | LC3 ↑  ATG7 ↑  p62 ↓  mTOR ↓ | Sprague-Dawley rats induced by streptozotocin (STZ) | up | (111) |
| **Monomer from Chinese Herb** | **Kaempferol**  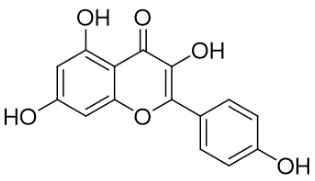 | Islet β cytoprotective effect | ATG7 ↑  ATG5 ↑  LC3B ↑  P62 ↓ | RIN-5F cell line | up | (63) |
|  | **Silibinin**  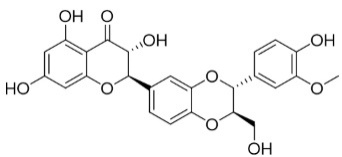 | Islet β cytoprotective effect | pAkt/Akt ↑ | INS-1 cells | up | (64) |
|  | **Resveratrol**  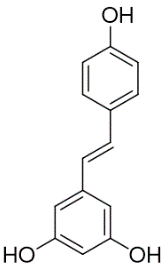 | DN | Atg 5 ↑  LC3-II/LC3-I ↑  p62 ↓ | diabetic db/db and db/m mice with a C57BL/KsJ genetic background | up | (113) |
|  |  | DC | FOXO1 ↑ | Wild-type male C57BL/6J mice 12 weeks old induced by intraperitoneal injection of STZ | up | (168) |
|  | **Quercitrin**  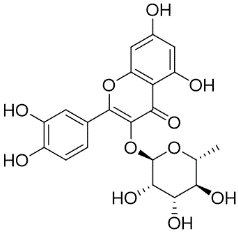 | Endothelial dysfunction | Autophagy ↑ | EPCs were isolated from mouse bone marrow | up | (167) |
|  |  | DR | Beclin-1 ↓  LC3-II/I ↓ | Human retinal microvascular endothelial cells (HRMECs) | down | (221) |
|  | **Triptolide**  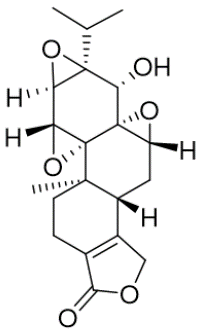 | DN | miR-141-3p/PTEN/Akt/mTOR pathway mediated autophagy ↑ | Six-week-old male Sprague-Dawley rats, HMCs | up | (114) |
|  | Astragaloside IV  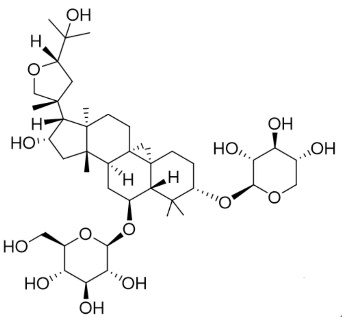 | DN | AMPKα ↑ | 6-week old male C57BL/6J mice, Mouse podocyte cell lines mouse^[137]^, glomerular MC line SV40 MES 13^[138]^ | up | (115， 116) |
|  |  | DH | AMPK ↑  mTOR ↓ | Male Sprague-Dawley rats (age, 6–8 weeks; weight, 200±20 g) | up | (142) |
|  | Ginsenoside Rg1  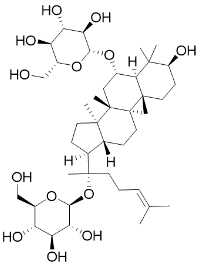 | DN | pAkt/Akt ↑  LC3-II/LC3-I ↑  p62 ↓  Beclin 1 ↑ | SPF-grade male Sprague-Dawley rats (aged 8 weeks,  weighing 180–200 g) | up | (117) |
|  | Ferulic Acid  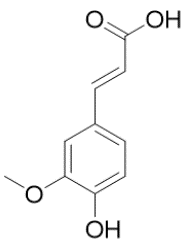 | DN | p62 ↓  LC3-II/LC3-I ↑  p-mTOR/mTOR ↓  pAMPK/AMPK ↑ | Male Wistar rats (adult, healthy of approximately 180–200 g  weight) | up | (112) |
|  | Punicalagin  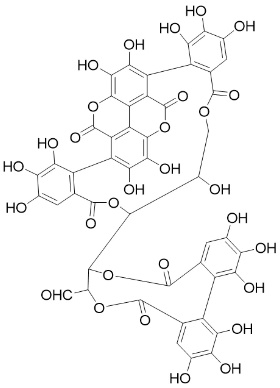 | DH | Akt/FoxO3a ↑ | male C57BL/6J mice (8−10  weeks old, 20 ± 2 g), HepG2 cell line | up | (141) |
|  | Carnosic Acid  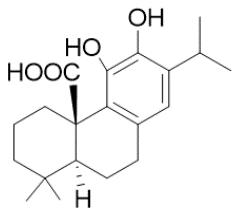 | DC | LC3-II/LC3-I ↑  p62 ↓ | Adult male C57BL/6 mice | up | (165) |
|  | β carotene  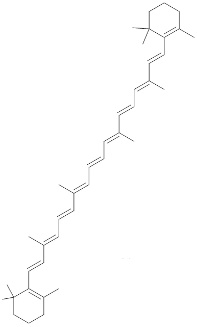 | DC | LC3-II/LC3-I ↓  p62 ↑ | H9c2 cells | down | (169) |
|  | Curcumin  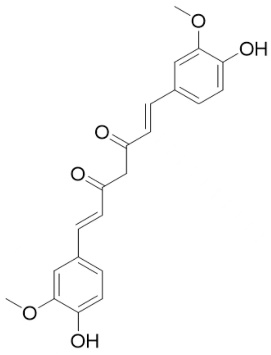 | DC | LC3-II/LC3-I ↑  p62 ↓ | Male C57BL/6 mice | up | (170) |
|  | Berberine  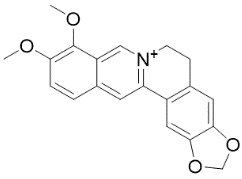 | DACD | AMPK ↑  mTOR ↓ | C57/BL6 mice (6–8 weeks of age, 18-22 g of body weight) | up | (202) |
|  | Gypenoside XVII | DR | LC 3II/I ↑  P62 ↓  Beclin 1 ↑  ATG5 ↑ | 20-week-old male db/db mice | up | (222) |
|  | Norkurarinone and isoxanthohumol | DR | LC 3II/I ↓  P62 ↑  Beclin 1 ↓  ATG5 ↓  PI3K/Akt/mTOR ↑ | human retinal microvascular endothelial cell | down | (223) |
|  | Procyanidin   | DR | LC3II/I ↓  p62 ↑  p-mTOR ↑ | human RPE cell line (ARPE-19) | down | (224) |
|  | (-)-epigallocatechin-3-gallate (EGCG), a polyphenol derived from green tea | DC | pAMPK ↓  LC3-II/LC3-I ↓  Atg 7 ↓ | Four-week-old male diabetic GK rats and age-matched male non-diabetic Wistar rats | down | (166) |
| **Experimental Chemicals** | 4-Phenylbutyric acid and rapamycin  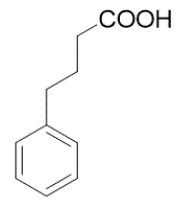 | Islet β cytoprotective effect | LC3BII ↑  ER stress-oxidative ↑ | male Wistar rats weighing  180–200 g | up | (65) |
| **Others** | Exercise | Islet β cytoprotective effect | AMPK ↑  PGC1aα ↑ | Eight-week-old male C57BL/6J mice | up | (66) |
